# Decreased eggshell strength caused by impairment of uterine calcium transport coincide with higher bone minerals and quality in aged laying hens

**DOI:** 10.1186/s40104-023-00986-2

**Published:** 2024-03-04

**Authors:** Yu Fu, Jianmin Zhou, Martine Schroyen, Haijun Zhang, Shugeng Wu, Guanghai Qi, Jing Wang

**Affiliations:** 1 Key Laboratory of Feed Biotechnology, Ministry of Agriculture and Rural Affairs, Institute of Feed Research, Chinese Academy of Agricultural Sciences, Beijing, 100081 China; 2 Precision Livestock and Nutrition Laboratory, Gembloux Agro-Bio Tech, TERRA Teaching and Research Centre, University of Liège, Gembloux, B-5030 Belgium

**Keywords:** Bone parameter, Calcium transport, Eggshell quality, Laying hen, Tissue damage

## Abstract

**Background:**

Deteriorations in eggshell and bone quality are major challenges in aged laying hens. This study compared the differences of eggshell quality, bone parameters and their correlations as well as uterine physiological characteristics and the bone remodeling processes of hens laying eggs of different eggshell breaking strength to explore the mechanism of eggshell and bone quality reduction and their interaction. A total of 240 74-week-old Hy-line Brown laying hens were selected and allocated to a high (HBS, 44.83 ± 1.31 N) or low (LBS, 24.43 ± 0.57 N) eggshell breaking strength group.

**Results:**

A decreased thickness, weight and weight ratio of eggshells were observed in the LBS, accompanied with ultrastructural deterioration and total Ca reduction. Bone quality was negatively correlated with eggshell quality, marked with enhanced structures and increased components in the LBS. In the LBS, the mammillary knobs and effective layer grew slowly. At the initiation stage of eggshell calcification, a total of 130 differentially expressed genes (DEGs, 122 upregulated and 8 downregulated) were identified in the uterus of hens in the LBS relative to those in the HBS. These DEGs were relevant to apoptosis due to the cellular Ca overload. Higher values of p62 protein level, caspase-8 activity, Bax protein expression and lower values of Bcl protein expression and Bcl/Bax ratio were seen in the LBS. TUNEL assay and hematoxylin-eosin staining showed a significant increase in TUNEL-positive cells and tissue damages in the uterus of the LBS. Although few DEGs were identified at the growth stage, similar uterine tissue damages were also observed in the LBS. The expressions of runt-related transcription factor 2 and osteocalcin were upregulated in humeri of the LBS. Enlarged diameter and more structural damages of endocortical bones and decreased ash were observed in femurs of the HBS.

**Conclusion:**

The lower eggshell breaking strength may be attributed to a declined Ca transport due to uterine tissue damages, which could affect eggshell calcification and lead to a weak ultrastructure. Impaired uterine Ca transport may result in reduced femoral bone resorption and increased humeral bone formation to maintain a higher mineral and bone quality in the LBS.

**Graphical Abstract:**

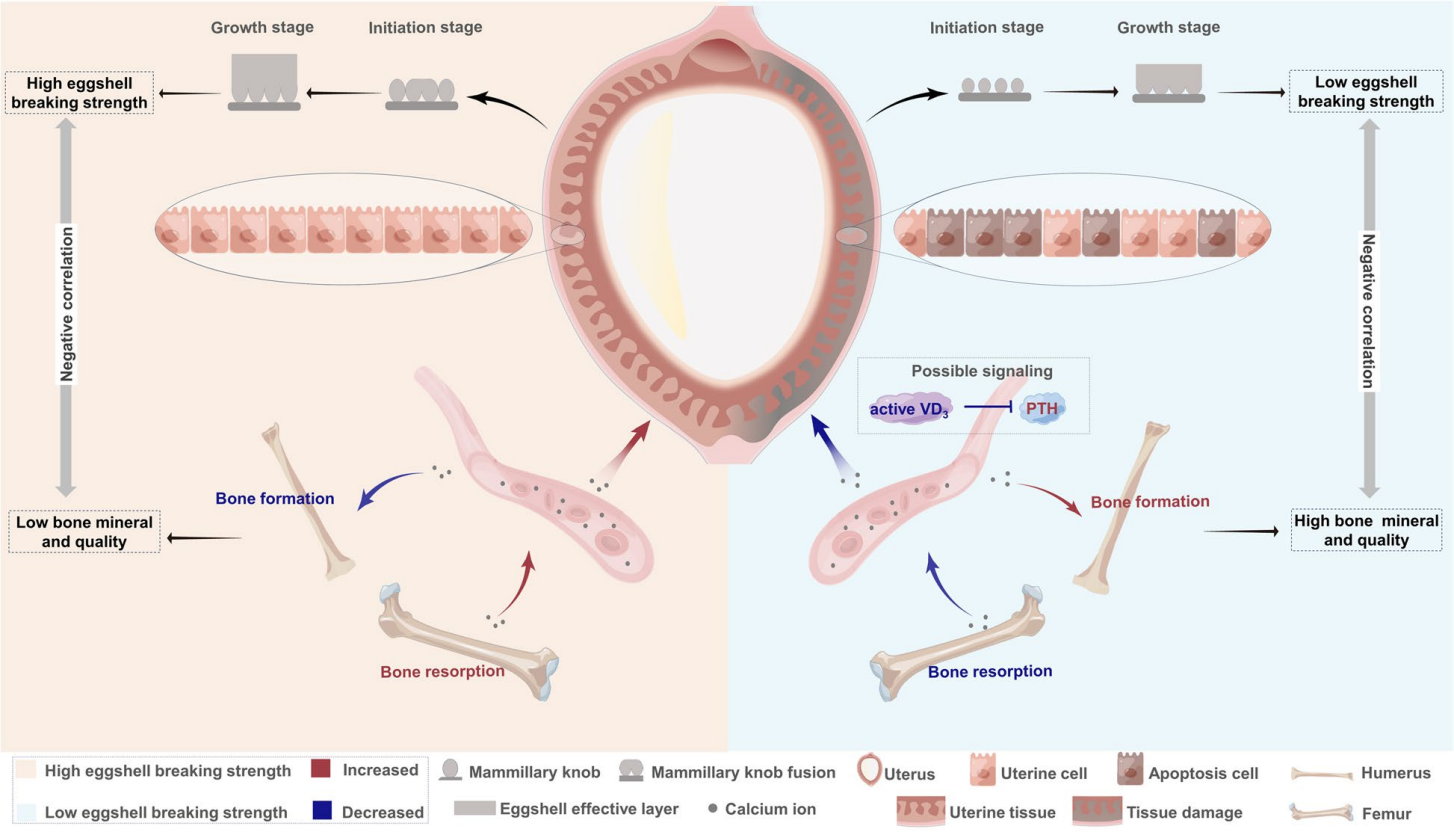

**Supplementary Information:**

The online version contains supplementary material available at 10.1186/s40104-023-00986-2.

## Introduction

An increase in cracked eggs in the late phase of the laying period heavily reduces the economic benefits of egg farmers and hinders the implementation of an extending laying period [1, 2]. Eggshell breaking strength is the ability of an eggshell to resist damage under external forces, and each decrement of 1 N breaking strength is associated with a 1.33% breakage in a flock with a high egg production (> 70%) [3]. As the age increased (30 to 80 weeks), the eggshell breaking strength declined from around 48 N to 35 N and its coefficients of variation increased from 11.4 to 17.2, leading to an increase in the proportion of individuals with low eggshell breaking strength in the Hy-line Brown laying hens [4, 5]. These hens laying weak-shelled eggs deserve special attention since they are responsible for a high breakage rate of eggs at the late laying period. Nutritional modulation strategies have been widely recognized as positive means of improving egg, especially eggshell, quality [6–9]. Comparison of eggshells and physiological characteristics of hens with high and low eggshell breaking strength could reveal the mechanism of increased egg breakage rate in aged laying hens, which is beneficial to develop more suitable products or measures for eggshell quality improvement.

Bone quality is receiving the same attention as eggshell quality in aged laying hens, and there may be a certain association between them since bone resorption provided approximately 20%−40% Ca for eggshell calcification [10, 11]. Medullary bone is considered as a Ca reservoir for eggshell calcification, which mainly exists within the marrow cavities of hind limbs (femur and tibia) in laying hens [12, 13]. Hens that did not lay eggs had more highly mineralized bones with significant amounts of medullary bone [14, 15], while high-laying hens underwent an increase in the bone fracture incidence due to Ca depletion during eggshell calcification [14]. Kim et al. [16] suggested that having poor-quality bones was linked to laying high-quality eggshells as high deposition of eggshell Ca was accompanied by high bone Ca transfer. Thus, the high Ca requirements of eggshell calcification may be a trigger for weakened bones, owing to intense medullary bone resorption [13, 17]. However, Alfonso-Carrillo et al. [3] concluded that the bone characteristics and eggshell properties were independent because they found no significant correlations between the eggshell (breaking strength, thickness and weight ratio) and bone (geometric and mechanical characteristics) quality of the hens at the end of laying period. Most previous studies were mainly concluded from limited skeletal indicators, presenting difficulties in determining the detailed correlations between bone and eggshell qualities as well as in analyzing the mechanisms underlying the effect of eggshell calcification on bone quality. The bone quality was determined by the bone remodeling, and more bone resorption and/or less bone formation contribute to a reduced bone mass and an increased incidence of bone fracture [18]. During bone formation, mesenchymal stem cells differentiate osteoblasts, which synthesize and secrete the organic matrix such as collagen type I (COL1), osteocalcin (OCN), osteopontin (OPN), bone sialoprotein (BSP) and osteonectin (ON), then deposit hydroxyapatite in the extracellular matrix and form mineralized bone, ultimately increasing bone mass [19, 20]. As for the process of bone resorption, bone minerals are dissolved and expose organic matrix to cathepsin K (Cts K) that degrades them, resulting in bone loss and structure damage [21, 22]. In the current study, we compared the bone quality and bone remodeling variation between hens with high and low eggshell breaking strength under the same feed and environment, which may contribute to a comprehensive understanding of the interaction mechanism of eggshell calcification and bone remodeling.

Eggshell formation takes about 18 h, and ultimately forms eggshell ultrastructure (including eggshell membrane, mammillary layer, effective layer (palisade layer and vertical crystal layer) and cuticle) to resist external forces. The mammillary layer and effective layer are major structures that determine an eggshell’s mechanical properties, which are respectively formed during the initiation (5–10 h post-ovulation) and growth (10–22 h post-ovulation) stages of eggshell calcification [23]. The eggshell Ca supply may have different adaptations due to the limitation of the photoperiod on feed access [24]. Generally, in the initiation stage of eggshell calcification, the Ca necessary is mainly derived from the intestinal absorption [24]. However, a considerable time of the growth stage of eggshell calcification typically occurs during the nocturnal fasting period [25]. At this period, medullary bone resorption becomes dominant in the supply for eggshell Ca as the residual Ca in the intestine is gradually consumed [24]. The increase in medullary bone resorption decreases bone minerals, induces osteoporosis, and affects the health of laying hens [26]. Previous research mainly targeted skeletons with medullary bone, such as tibia and femur [3, 27]. The tibia is considered as a model bone for the skeletal health examination in poultry species, while the femur is the most labile source of medullary bone Ca [28, 29], thus the femur may be more suitable to explore the function of bone with medullary bone during eggshell calcification. Additionally, birds retained a special type of bones during evolution, that is a hollow bone such as the humerus adapted to fly [30]. Humerus and femur may be remodeled in a different manner that affect eggshell calcification due to a differential sensitivity to hormones [30, 31]. Exploring the metabolic characteristics of different bones during eggshell calcification could provide a more comprehensive panorama of the bone remodeling response to eggshell calcification.

This study compared the eggshell quality and bone parameters of hens with high or low eggshell breaking strength to analyze the correlations between eggshell and bone qualities of aged hens. Furthermore, their uterine transcription profiles, histological characteristics and bone remodeling processes during eggshell calcification were explored to reveal the possible mechanism of eggshell and bone quality reduction and the interaction of the uterus and skeletons of aged hens. This study provides some new insights into the crosstalk between bone and uterus, which is conducive to explore the regulation of bone and eggshell quality.

## Materials and methods

### Birds and experiment design

**Fig. 1:**
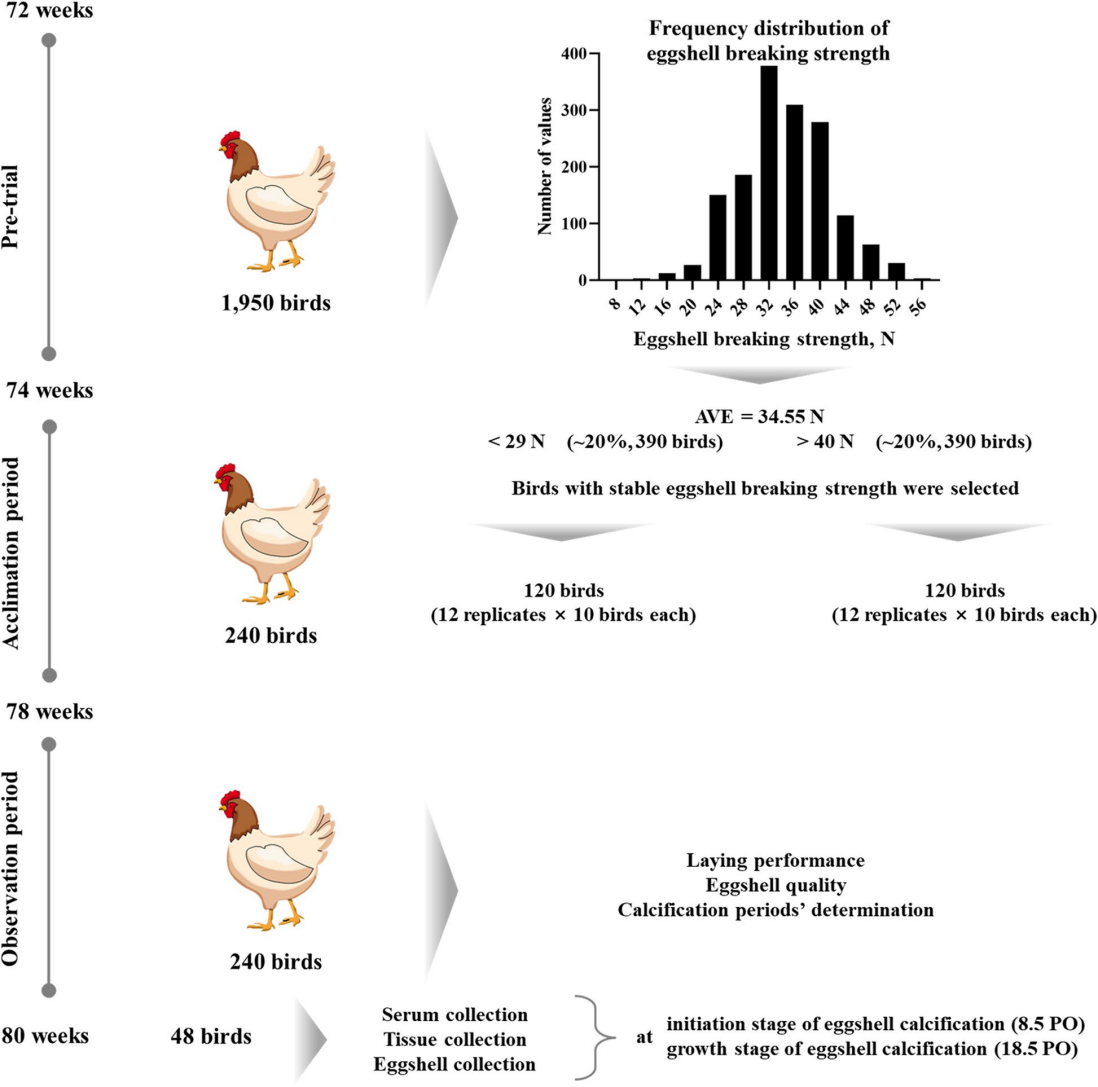
Trial design and sampling scheme. PO, post-oviposition

Animal procedures were approved by the management of the Animal Care and Use Committee of Institute of Feed Research, Chinese Academy of Agricultural Sciences (approval No. AEC-CAAS-20200902). The present study design is depicted in Fig. 1. A total of 1,950 72-week-old healthy Hy-line Brown laying hens were caged individually, and the eggshell breaking strength of each hen was measured daily during a 2-week pre-trial period. The average egg production of this flock was 88.79%. The average eggshell breaking strength of the flock was 34.55 N, in which the hens with average breaking strength above 40 N and below 29 N accounted for 20%, respectively. After the pre-trial, 240 laying hens were selected and divided into high (> 40 N, HBS) and low (< 29 N, LBS) eggshell breaking strength groups according to eggshell breaking strength. The egg production of the selected hens complied with 88.79% ± 5%, and their egg weight was in accordance with 55 to 70 g. The hens that were not selected were still raised in the commercial hen house until elimination. The selected hens were transferred to another hen house with the same management, and each group was sub-divided into 12 replicates with 10 hens each. All hens were caged individually and fed with the same basal diet (Additional file 1; Ca level: 3.89%, the ratio of Ca and total P: 9.05:1) and received water ad libitum through the whole trial. All hens underwent an acclimation period of 4 weeks and an observation period of 2 weeks. During the observation period, the oviposition time of each hen was recorded daily using an automatic-monitoring control system (IFR, CAAS, Beijing, China) to determine the calcification periods [32]. All hens were subjected to a controlled photoperiod cycle of 16 h light:8 h dark (light: 5:30−21:30).

### Sample collection

A total of 25 eggs from each replicate were collected on 5 consecutive days of the observation period (5 eggs/replicate/d) to detect eggshell measurements. At 80 weeks of age, 12 birds (1 bird per replicate) in each group were selected at both 8.5 h post-oviposition (PO) and 18.5 h PO, corresponding to the initiation and growth stages of eggshell calcification, respectively. The selected hens were collected blood samples first, then subjected to euthanasia and tissue sampling. Serum was immediately separated and stored at −80 °C after blood collection. The uterus morphology and the position of the egg were observed before the tissue was removed. Then uterus tissues were quickly removed and placed on ice. The uterine mucosa was removed immediately and stored at −80 °C until further analysis. An additional piece of uterine tissue was fixed in the 4% paraformaldehyde solution for uterine histomorphology. The egg was carefully transferred from the uterus, and the eggshell was slowly isolated from the egg and dried naturally at room temperature. The humerus and femur on both sides of each bird were removed, and residual tissues were cleared. The right bones, after removing the cartilage, were stored at −80 °C until analysis. The left bones sampled at 18.5 h PO were truncated in the middle of the bone mid-diaphysis, with the proximal parts fixed in formalin. The remaining left bones were frozen at −20 °C for bone geometrical, mineral and compositional analysis.

### Laying performance

During the observation period, egg number and egg weight were recorded daily by replicate, and total feed consumption for each replicate was weighed. The egg production rate, egg mass, average egg wight, average daily feed intake and feed conversion ratio were calculated. Egg production rate was calculated as the ratio of the number of eggs produced per day to the number of birds, expressed as a percentage. Egg mass was represented as the weight of egg production per laying hen per day. Feed conversion ratio was calculated as grams of total feed consumption/total egg weight for each replicate.

### Eggshell quality

#### Eggshell mechanical properties

Eggshell quality (2 groups, 12 replicates per group, 25 eggs per replicate) was determined according to the methods as described earlier [33]. The egg weight was weighed first. Then, the eggshell thickness was tested with the Egg Shell Thickness Gauge (Israel Orka Food Technology Ltd., Ramat Hasharon, Israel) and calculated as the mean of equator and both poles of the egg. Afterwards, eggshell breaking strength was detected by the Egg Force Reader (Israel Orka Food Technology Ltd., Ramat Hasharon, Israel). After removing the egg content, the eggshell was washed, dried and weighed. The eggshell weight ratio was calculated as the percent ratio of the eggshell weight over the total egg weight.

#### Eggshell ultrastructure

Five eggshells per replicate (collected on the observation period) were collected to assess eggshell ultrastructure (in total 60 eggshells per group). The eggshell was washed with distilled water to remove the dirt and residual albumen. After drying at room temperature, two pieces (~ 0.5 cm^2^ each) of each eggshell were taken from the equatorial region, fixed in the specimen stage, and coated with gold powder. The vertical profiles of eggshells were imaged using a scanning electronic microscopy (SU8020, Hitachi Co., Ltd., Tokyo, Japan). The thickness of effective and mammillary layers was measured with the SEM ruler according to a previous report [32]. Briefly, the mammillary thickness was assessed by measuring the length from the top of the membrane to the lower edge of the palisade layer. The effective thickness was taken as the length from the top of the cuticle to the bottom of the palisade layer. The calcified layer referred to the combined effective and mammillary layers. The thickness ratio was defined as the percentage of each layer relative to the calcified layer. Each sample was measured 3 times at random. Two pieces of each eggshell, which were obtained at 8.5 h PO, were fixed at an aluminum plate to observe their vertical and external surfaces. Eggshells (two pieces with ~ 0.5 cm^2^ each) collected at 18.5 h PO were similarly fixed to photograph the vertical profile. The thickness of effective, mammillary and calcified layers was determined as described above.

#### Eggshell components

Five eggshells per replicate, weighing close to the average eggshell weight, were collected on the observation period. The eggshells were first washed, dried and weighed (*W*_1_). Then, equal weights from each eggshell were taken, mixed and crushed into one sample for the determination of eggshell Ca and P content according to a previously reported method [33]. Briefly, 0.5 g of eggshell power was dissolved in 3 mL hydrogen peroxide and 3 mL nitric acid then withheld for 2 h. A microwave digestion system (MDS-10, Shanghai Xinyi Instrument Technology Co., Ltd., Shanghai, China) was used to further digest the samples. The Ca and P contents were determined as *C*_1_ and *C*_2_ by a flame atomic absorption spectrophotometry (Z2000, Hitachi Co., Ltd., Tokyo, Japan) and a spectrophotometer (UV-2700, Shimadzu Corp., Kyoto, Japan), respectively. Total Ca and P per eggshell were calculated using the following formula: total Ca per eggshell = *W*_1_/5×*C*_1_, total P per eggshell = *W*_1_/5×*C*_2_.

### Bone quality

#### Bone geometrical characteristics

The left bones (1 bone per replicate) sampled at 8.5 h PO were thawed at 4 °C overnight and equilibrated at room temperature for 2 h. The whole bone was weighed (*W*_2_) first, then it was placed in a measuring cylinder with some water and the increased volume was recorded as bone volume (*V*). The density of bone was calculated according to the following formula: *W*_2_/*V*. The length and midpoint perimeter of the bone were measured using a string and a digital caliper. A digital caliper was used to determine the diameters of the incision site of the distal left bones collected at 18.5 h PO. The external and internal cortical bone diameters were defined as H and h in the horizontal (medial-lateral) plane and as B and b in the vertical (anterior-posterior) plane. The cortical cross-sectional area, mean relative wall thickness and mean cortical index were calculated with equations as previously described [34, 35]: cortical cross-sectional area = π × [(H × B) − (h × b)]/4; mean relative wall thickness = [(B − b)/b + (H − h)/h]/2; mean cortical index = [(B − b)/B + (H − h)/H]/2.

#### Bone mineral measurements

After the analysis of geometrical characteristics, the left bones (1 bone per replicate, sampled at 8.5 h PO) without any soft tissues were used to determine bone mineral content (BMC) and bone mineral density (BMD) with a dual energy X-ray absorptiometry (DTX-200, Osteometer MediTech, Hawthorne, CA, USA) according to previous studies [31, 36]. The air was used to calibrate the measurements. Three regions were tested: the proximal, middle and distal sections, each of which was 1 cm long. The detection regions were kept consistent for all samples.

#### Bone components

The bone (1 sample per replicate), after measuring BMC and BMD, was used to determine bone components. The bone was broken first, and at each subsequent step, all bone fragments from each sample were carefully collected to ensure that all measurements were conducted on the whole bone. The samples were dehydrated in ethanol and defatted with petroleum ether, followed by drying in the oven overnight at 105 °C. The fat-free dry bone was weighed. Then, the determination of bone ash was carried out using a muffle furnace. The ash content of the bone sample was calculated via division of the ash weight by the fat-free dry weight. The bone Ca and P contents in ash were measured as described by the method above (“Eggshell Quality, Ultrastructure and Components”), followed by calculating the ratio of Ca and P. Total Ca/P per bone was the product of bone Ca/P content in ash and ash weight.

#### Bone histomorphometry

The method of Goldner’s Trichrome stain was carried out as previously reported [37]. The formalin-fixed specimens (1 sample per replicate) were decalcified, dehydrated and embedded in paraffin. A microtome was used to slice the paraffin blocks into the sections that were 4 μm thick. After dewaxing, Goldner’s Trichrome staining was performed with a commercial kit (Servicebio technology Co., Ltd., Wuhan, China) according to the manufacturer’s instructions. Pannoramic scanner system (3DHISTECH Ltd., Budapest, Hungary) was used to scan the images of the bones.

### RNA extraction, library construction, sequencing and data analysis in the uterus

Total RNA of the uterus samples was extracted with TRNzol reagent (Tiangen Biotech Co., Ltd., Beijing, China), and its integrity was verified by the Agilent Bioanalyzer 2100 system (Agilent Technologies, CA, USA). RNA Libraries were prepared using NEBNext^®^ Ultra™ RNA Library Prep kit for Illumina^®^ (NEB Inc., Ipswich, MA, USA). Briefly, the mRNA was enriched using magnetic beads (for eukaryotes) with Oligo (dT) and broken into short fragments of approximately 150 bp in fragmentation buffer. Fragmented mRNA was used as a template, and first-strand cDNA was achieved using random hexamers. The second strand cDNA was subsequently synthesized by adding dNTPs, RNase H, DNA polymerase I and buffer. The double-stranded cDNA was purified with AMPure XP beads, then terminal repair and 3′-end single nucleotide A (adenine) addition were performed for adaptor ligation. Fragment size selection of library was also carried out using AMPure XP beads. The cDNA library was constructed by PCR amplification. AMPure XP system (Beckman Coulter, Beverly, USA) was used for the purification of PCR products, and Agilent Bioanalyzer 2100 system was used to assess library quality. Finally, the TruSeq PE Cluster kit v4-cBot-HS (Illumina, San Diego, CA, USA) was used to perform the clustering of the index-coded samples on a cBot Cluster Generation System, and the libraries were sequenced on an Illumina platform (Illumina, San Diego, CA, USA). The sequence data have been submitted to the NCBI Sequence Read Archive under the accession numbers: PRJNA1000560.

The raw reads were filtered by removing low-quality reads with ambiguous ‘N’ base, adapter sequences, rRNA, and short reads (less than 20 nt) with FasTX clipper v0.0.13. The resulting clean reads were mapped using HISAT2 [38] with *Gallus gallus* *GRCg6* as the reference annotation file. The expression levels of mapped genes were normalized as FPKM using StringTie. The differentially expressed genes (DEGs) between the HBS and LBS groups were analyzed separately at 8.5 and 18.5 h PO. The analysis of DEGs was performed using the DESeq2 software, with the following screening threshold: |Fold change| > 1.3 and the false discovery rate (FDR, adjusted with Benjamini and Hochberg’s approach) < 0.05. The DEGs were mainly obtained from 8.5 h PO, while there were only a few differences of transcriptional profiles at 18.5 h PO between HBS and LBS. Thus, only DEGs at 8.5 h PO were used for onward analysis. To elaborate on the activated pathways associated with the differences of mammillary knobs’ growth at 8.5 h PO, we analyzed for enriched Gene Ontology (GO) biological processes on DEGs using ClueGO.

### qRT-PCR validation of RNA sequencing results

Twelve genes of each eggshell calcification stage were selected for qRT-PCR validation. The cDNA was synthesized using reverse transcription with Easy Script First-Strand cDNA Synthesis SuperMix kit (TransGen Biotech Co., Ltd., Beijing, China) following manufacturer’s instruction. Each reverse transcription included 1.5 µg RNA. Quantitative PCR assays were performed utilizing a CFX96 C1000TM thermal cycler (Bio-Rad, CA, USA). And each assay was done with 3 technical replicates. Primer sequences are listed in Additional file 2. Primer efficiencies ranged from 91.08% to 109.70%.

### Apoptosis-related markers in the uterus

The p62, p53, LC3 and immunoglobulin A protein contents of the uterus were quantified using an ELISA method. The kits of p62 and p53 were purchased from Shanghai Enzyme-linked Biotechnology Co., Ltd. (Shanghai, China), and those of LC3 and immunoglobulin A were obtained from Jiangsu Meimian Industrial Co., Ltd. (Jiangsu, China). The activities of caspase-3 and caspase-8 were assessed using the caspase-3 and caspase-8 activity assay kits (Beyotime, Shanghai, China) according to the manufacturer’s protocol. The protein concentrations were measured with a Bradford protein assay kit (Beyotime).

Western blot analysis was performed to detect the relative expression of Bcl-2 and Bax proteins. Total proteins were extracted by a commercial kit (Beyotime), supplemented with protease/phosphatase inhibitors (Beyotime). Lysis buffer contained 20 mmol/L Tris (pH 7.5), 150 mmol/L NaCl, 1% Triton X-100 and a cocktail of protease inhibitors. The protein concentrations were measured with a BCA protein assay kit (Beyotime). A total of 20 µg protein was loaded in each lane. Following electrophoresis, the protein samples were transferred to a PVDF membrane (Bio-Rad, CA, USA). The membrane was blocked with 5% non-fat dry milk (TBS solution with 0.1% Tween) for 45 min with agitation at room temperature, followed by overnight incubation with primary antibodies for Bax (Abclonal, #A12009), Bcl-2 (Abclonal, #A11025) and GAPDH (Abcam, #EPR16891), respectively. The membrane was then washed 3 times for 10 min each time, and incubated with secondary antibody for 1 h at room temperature. After washing membrane again, the blots were visualized with ECL reagent (Beyotime) in a dark room. Image analysis was conducted using Image-Pro Plus 6.0 software.

### Hematoxylin-eosin (HE) and TUNEL staining

Tissue samples of the uterus were fixed in formalin overnight and embedded in paraffin blocks. The blocks were processed for routine microtome and stained by HE for histopathological observation. Another section was used to assay apoptosis with a fluorescein TUNEL assay kit (G1501, Servicebio Technology Co., Ltd., Hubei, China) based on manufacturer’s instruction. Firstly, tissue sections were deparaffinized and rehydrated. Proteinase K solution was then added to retrieve the antigen. Subsequently, membranes were disrupted, the TUNEL reaction solution was added, and the nuclei were stained with DAPI solution. Finally, microscopic examination and image collection were conducted using a fluorescence microscope (Nikon Instruments Inc., Tokyo, Japan). Blue color indicates cell nucleus, and green color indicates positive apoptosis cells.

### Quantification of bone remodeling-related mRNA in bone

The humerus and femur samples without cartilage were removed from −80 °C. The sample was hammered into pieces and further ground manually with a mortar and pestle in the liquid nitrogen. Total RNA was extracted using EASYspin Plus Bone Tissue RNA kit (Aidlab Biotechnologies Co., Ltd., Beijing, China) according to the kit instructions. Agarose gel electrophoresis was used to confirm RNA integrity, and a NanoDrop 2000 spectrophotometer (Thermo Fisher Scientific, Waltham, MA, USA) was used to determine the purity and concentration of the RNA. The cDNA synthesis and quantification of target gene expression were performed as described in the “qRT-PCR validation of RNA sequencing results”. The primers are also supplemented in Additional file 2. Primer efficiencies ranged from 91.91% to 107.86%.

### Calcium, phosphorus, bone remodeling-related enzyme and hormone concentrations in serum

Serum was thawed at 4 °C and analyzed for Ca and P concentrations using a microplate reader with Ca and P assay kits (Nanjing Jiancheng Bioengineering Institute, Nanjing, China). The bone-specific alkaline phosphatase (BALP) level was detected using a commercial kit (Shanghai Meilian biological Technology Co., Ltd., Shanghai, China), and the tartrate resistant acid phosphatase (TRAP) activity were determined using a TRAP activity kit (Shanghai Meilian biological Technology Co., Ltd.). Radioimmunoassay (RIA) kits were purchased from Beijing Sino-UK Institute of Biological Technology (Beijing, China) to determine parathyroid hormone (PTH), estrogen (E_2_), 25-hydroxyvitamin D_3_ (25(OH)D_3_) and 1,25-dihydroxyvitamin D_3_ (1,25(OH)_2_D_3_) concentrations in serum.

### Statistical analysis

Replicates (*n* = 12) were the experimental units for all analysis. The normal distribution (Shapiro-Wilk test) and homoscedasticity (Levene’s test) of all data were firstly checked using SAS 9.4 (SAS Inc., Cary, NC, USA). An unpaired two-tailed Student’s *t*-test was also conducted in SAS 9.4 (SAS Inc., Cary, NC, USA) to analyze the significance of the difference between the HBS and LBS. Data are presented as mean ± SD (standard deviation). Differences were considered significant at a *P-*value < 0.05. The correlation between eggshell and bone qualities was analyzed using Origin software. Relative gene expression levels were calculated using the 2^−∆∆Ct^ method and normalized to avian β-actin as housekeeping gene.

## Results

### Laying performance and eggshell quality

The results of laying performance as well as eggshell quality and components are shown in Table 1. No significant differences were found in the laying performance (egg production rate, egg mass, average egg weight, average daily feed intake and feed conversion ratio) between HBS and LBS (*P* > 0.05). The eggshell breaking strength, thickness, weight and weight ratio were significantly lower in the LBS than those in the HBS (*P* < 0.05). HBS and LBS showed no significant difference in Ca and P contents of eggshell (*P* > 0.05). However, total Ca per eggshell and total P per eggshell were significantly reduced in the LBS (*P* < 0.05).

**Table 1:** Differences in laying performance and eggshell qualities of the hens laying eggs with different eggshell breaking strength^a^

Items	HBS	LBS	*P*-value
Laying performance			
Egg production rate, %	89.35 ± 2.75	86.37 ± 3.88	0.060
Egg mass, g/hen/d	56.36 ± 2.54	55.36 ± 3.76	0.456
Average egg weight, g	63.19 ± 1.69	64.16 ± 2.30	0.250
Average daily feed intake, g/hen/d	107.74 ± 6.31	105.43 ± 5.78	0.360
Feed conversion ratio, g/g	1.92 ± 0.16	1.91 ± 0.15	0.938
Eggshell quality			
Breaking strength, N	46.79 ± 2.56	25.99 ± 2.51	< 0.001
Eggshell thickness, mm	0.35 ± 0.02	0.30 ± 0.03	< 0.001
Eggshell weight, g	6.59 ± 0.42	5.85 ± 0.41	< 0.001
Eggshell weight ratio, %	10.42 ± 0.42	9.13 ± 0.54	< 0.001
Eggshell components			
Ca content, mg/g	374.64 ± 7.32	378.27 ± 7.67	0.229
Total Ca per eggshell, g	2.47 ± 0.10	2.19 ± 0.08	< 0.001
P content, mg/g	0.96 ± 0.09	0.94 ± 0.11	0.619
Total P per eggshell, mg	6.29 ± 0.57	5.49 ± 0.73	0.007

*HBS *High eggshell breaking strength group, *LBS *Low eggshell breaking strength group

^a^Data represent means with standard deviation based on 12 replicates (10 birds/replicate for laying performance; 25 eggs/replicate for eggshell quality; 5 eggshells for eggshell components)

**Fig. 2:**
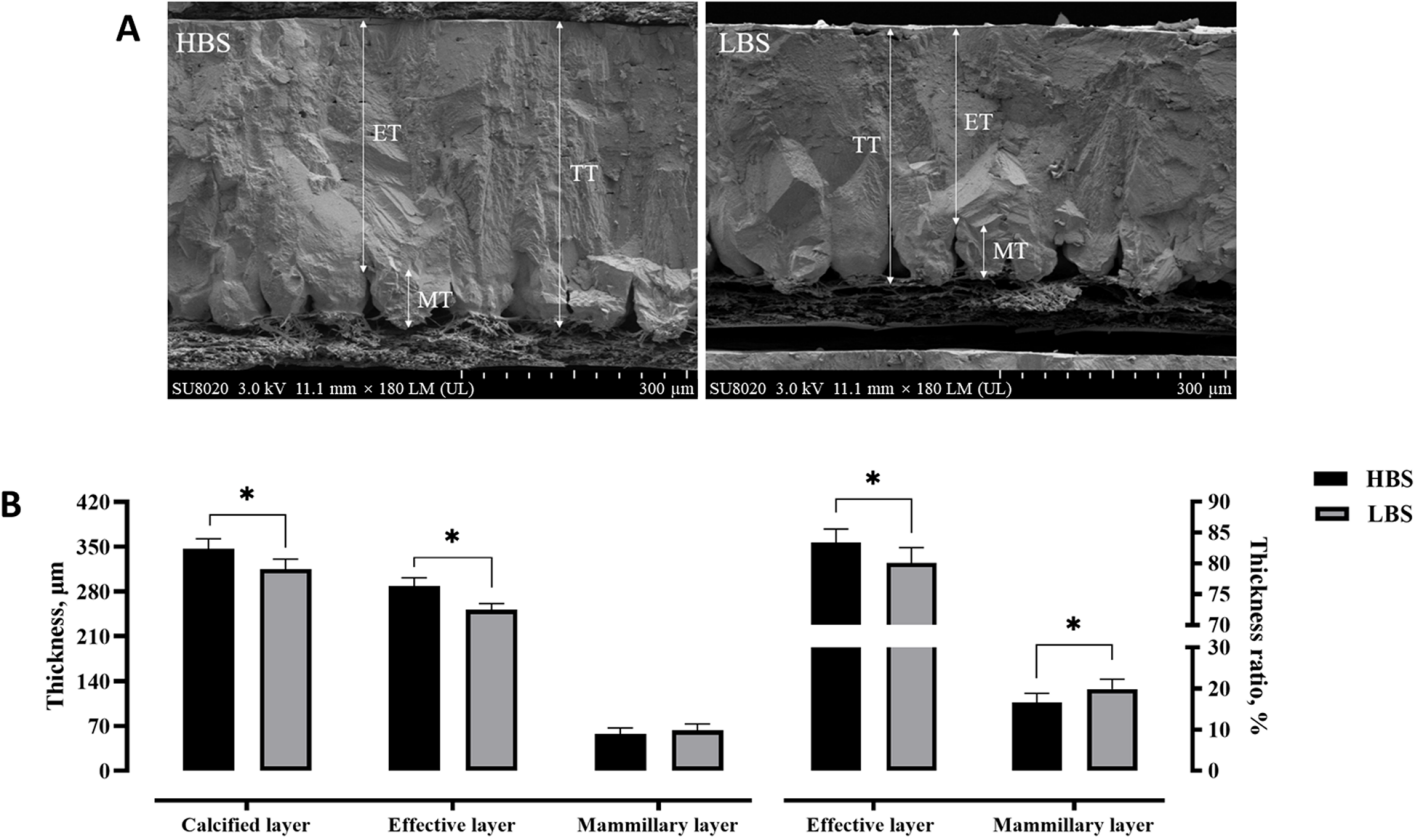
Differences in the eggshell ultrastructure of hens laying eggs with different eggshell breaking strength. **A** Images of eggshell vertical profiles in high (HBS) and low (LBS) eggshell breaking strength groups under a scanning electron microscope. **B** Results of the eggshell ultrastructural characteristics. TT, total thickness; ET, effective layer thickness; MT, mammillary layer thickness. Data represent means with standard deviation based on 12 replicates with 5 eggshells each. An asterisk (*) indicates a significant difference (*P* < 0.05) between groups

Scanning electron microscopy images in Fig. 2A show the eggshell ultrastructure of laying hens in HBS and LBS. Compared with the HBS, the LBS had a thinner thickness of calcified layer and effective layer (Fig. 2B, *P* < 0.05). The LBS significantly decreased the thickness ratio of effective layer while increasing that of mammillary layer (Fig. 2B, *P* < 0.05).

### Bone quality

The bone geometrical characteristics are presented in Table 2. The mean cortical index of both humeri and femurs were significantly higher in the LBS than in the HBS (*P* < 0.05). The mean relative wall thickness of femur in the LBS was thicker than that of the HBS (*P* < 0.05). No significant differences were observed between the two groups on the bone length, weight, volume, density, midpoint perimeter and cortical cross-sectional area (*P* > 0.05).

**Table 2:** Differences in bone geometrical characteristics of the hens laying eggs with different eggshell breaking strength^a^

Items	HBS	LBS	*P*-value
Humerus			
Length, cm	7.96 ± 0.25	7.93 ± 0.14	0.712
Weight, g	3.13 ± 0.09	3.36 ± 0.54	0.234
Volume, cm^3^	5.14 ± 0.71	5.21 ± 0.31	0.802
Density, g/cm^3^	0.62 ± 0.09	0.64 ± 0.09	0.554
Midpoint perimeter, cm	2.17 ± 0.14	2.08 ± 0.06	0.085
Cortical cross-sectional area, mm^2^	9.08 ± 0.80	9.91 ± 1.20	0.079
Mean relative wall thickness	0.18 ± 0.02	0.22 ± 0.07	0.061
Mean cortical index	0.15 ± 0.02	0.18 ± 0.04	0.045
Femur			
Length, cm	8.65 ± 0.09	8.78 ± 0.06	0.204
Weight, g	9.05 ± 0.27	9.11 ± 0.14	0.829
Volume, cm^3^	7.16 ± 0.26	7.21 ± 0.12	0.875
Density, g/cm^3^	1.27 ± 0.02	1.27 ± 0.02	0.976
Midpoint perimeter, cm	2.61 ± 0.06	2.68 ± 0.03	0.313
Cortical cross-sectional area, mm^2^	12.11 ± 2.25	13.99 ± 2.59	0.087
Mean relative wall thickness	0.15 ± 0.03	0.18 ± 0.04	0.042
Mean cortical index	0.13 ± 0.02	0.15 ± 0.02	0.047

*HBS* High eggshell breaking strength group, *LBS* Low eggshell breaking strength group

^a^Data represent means with standard deviation based on 12 replicates with 1 bird each

Table 3 compares bone mineral measurements between HBS and LBS. The humeral midshaft and proximal BMD of the LBS were significantly greater than those of the HBS (*P* < 0.05). The humerus in the LBS had significantly higher proximal BMC compared with that in the HBS (*P* < 0.05). However, there were no significant difference on mineral measurements of the femur between these two groups (*P* > 0.05).

**Table 3:** Differences in bone mineral measurements of the hens laying eggs with different eggshell breaking strength^a^

Items	HBS	LBS	*P*-value
Humerus			
Distal BMD, g/cm^2^	2.83 ± 0.18	2.96 ± 0.20	0.096
Midshaft BMD, g/cm^2^	2.89 ± 0.22	3.14 ± 0.30	0.028
Proximal BMD, g/cm^2^	2.68 ± 0.22	2.99 ± 0.22	0.002
Distal BMC, g	2.57 ± 0.14	2.62 ± 0.17	0.436
Midshaft BMC, g	1.66 ± 0.19	1.73 ± 0.19	0.366
Proximal BMC, g	2.35 ± 0.20	2.59 ± 0.18	0.005
Femur			
Distal BMD, g/cm^2^	2.75 ± 0.21	2.86 ± 0.19	0.218
Midshaft BMD, g/cm^2^	3.04 ± 0.26	3.04 ± 0.35	0.959
Proximal BMD, g/cm^2^	2.78 ± 0.26	2.80 ± 0.19	0.876
Distal BMC, g	2.50 ± 0.18	2.55 ± 0.17	0.551
Midshaft BMC, g	1.74 ± 0.14	1.68 ± 0.20	0.354
Proximal BMC, g	2.44 ± 0.22	2.41 ± 0.18	0.732

*HBS* High eggshell breaking strength group, *LBS* Low eggshell breaking strength group, *BMD* Bone mineral density, *BMC* Bone mineral content

^a^Data represent means with standard deviation based on 12 replicates with 1 bird each

Differences in the bone components between the HBS and LBS are presented in Table 4. The LBS had the humeri with higher fat-free dry weight, ash, organic matter, total Ca and P per bone in comparison with the HBS (*P* < 0.05). The ash and ash content of the femur were significantly higher in the LBS compared with those in the HBS (*P* < 0.05), while Ca and P contents in ash were lower (*P* < 0.05).

**Table 4:** Differences in bone components of the hens laying eggs with different eggshell breaking strength^a^

Items	HBS	LBS	*P*-value
Humerus			
Fat-free dry weight, g	2.24 ± 0.12	2.53 ± 0.30	0.021
Ash, g	1.39 ± 0.08	1.55 ± 0.17	0.022
Ash content, %	62.07 ± 0.99	61.22 ± 1.66	0.203
Organic matter, g	0.85 ± 0.05	0.98 ± 0.14	0.016
Ca content in ash, %	36.75 ± 0.35	37.19 ± 0.57	0.137
Total Ca per bone, mg	513.59 ± 17.49	586.49 ± 62.02	0.020
P content in ash, %	16.28 ± 0.22	16.47 ± 0.31	0.249
Total P per bone, mg	227.49 ± 7.77	259.75 ± 28.35	0.023
Ratio of Ca and P	2.25 ± 0.01	2.26 ± 0.03	0.916
Femur			
Fat-free dry weight, g	5.32 ± 0.53	5.70 ± 0.52	0.099
Ash, g	3.00 ± 0.35	3.40 ± 0.43	0.025
Ash content, %	56.33 ± 3.17	59.45 ± 2.64	0.021
Organic matter, g	2.32 ± 0.29	2.30 ± 0.14	0.848
Ca content in ash, %	37.41 ± 0.37	36.63 ± 0.60	0.022
Total Ca per bone, mg	1113.62 ± 94.55	1235.27 ± 137.7	0.105
P content in ash, %	16.48 ± 0.17	16.07 ± 0.29	0.012
Total P per bone, mg	490.46 ± 40.33	541.34 ± 54.71	0.097
Ratio of Ca and P	2.27 ± 0.01	2.28 ± 0.03	0.509

*HBS* High eggshell breaking strength group, *LBS* Low eggshell breaking strength group

^a^Data represent means with standard deviation based on 12 replicates with 1 bird each

**Fig. 3:**
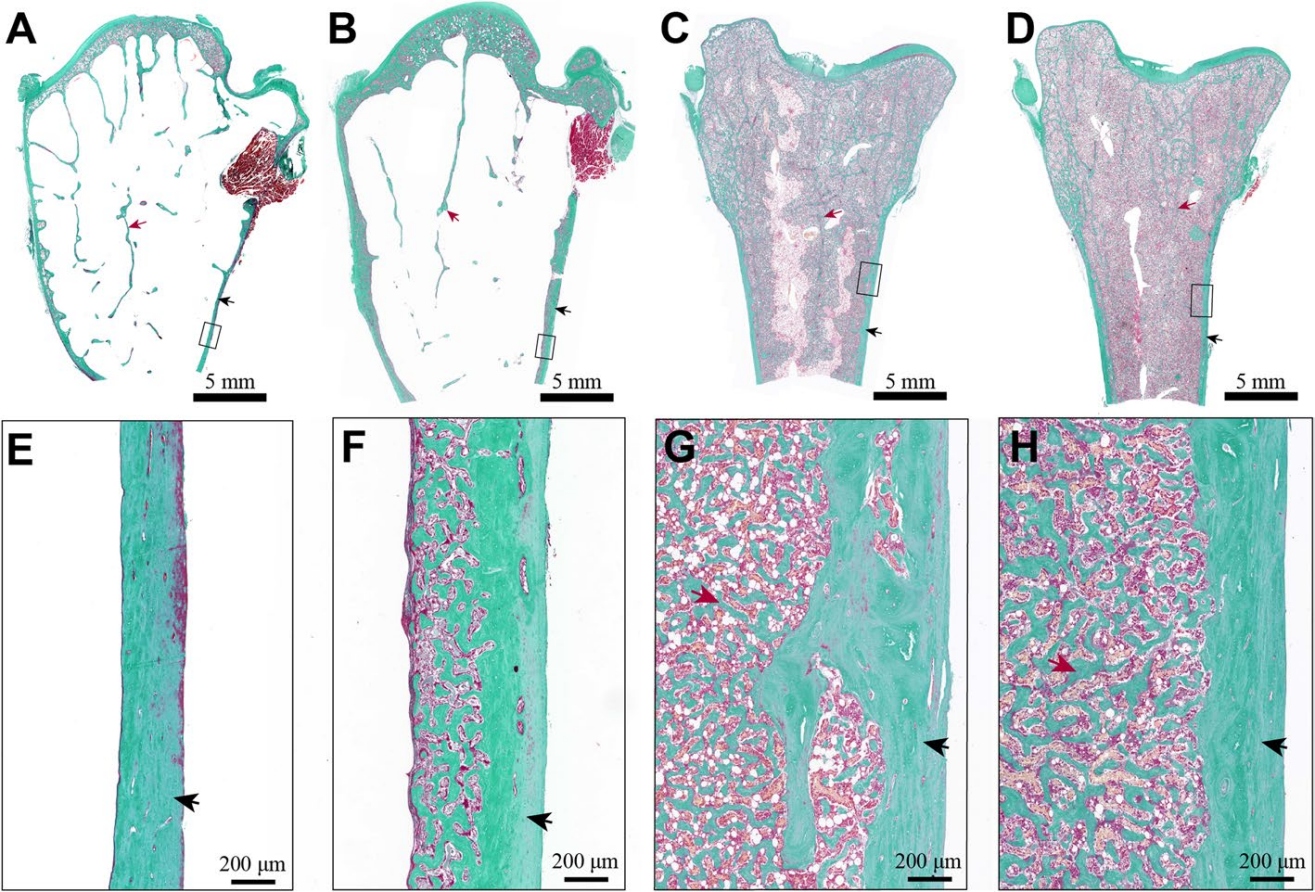
Differences in bone histomorphometry of the hens laying eggs with different eggshell breaking strength. The insets (**E–H**) are the zoomed-in images of the black boxes in **A–D**. (**A**,** B**,** E**,** F**) in the humerus; (**C**,** D**,** G**,** H**) in the femur; (**A**,** E**,** C**,** G**) in the HBS; (**B**,** F**,** D**,** H**) in the LBS. Black arrows, cortical bones; red arrows, trabecular bones. HBS, high eggshell breaking strength group; LBS, low eggshell breaking strength group

Representative images of the humerus and femur stained with Goldner’s Trichrome are illustrated in Fig. 3. The lateral edge of the cortical bone was clear and flat in both groups, whereas its medial margin in the femur had indistinct borders with adjacent trabecular bone. The cortical thickness of both femur and humerus was thicker in the LBS than in the HBS. The medial edge of the humerus in the LBS was attached by a thick layer of spongy bone, while no similar structure was observed in the HBS. More irregular erosions and demineralized regions were seen in the femoral intracortical region in the HBS. In contrast, the endocortical surface was flatter in the femur of the LBS.

### Correlations between eggshell and bone qualities

**Fig. 4:**
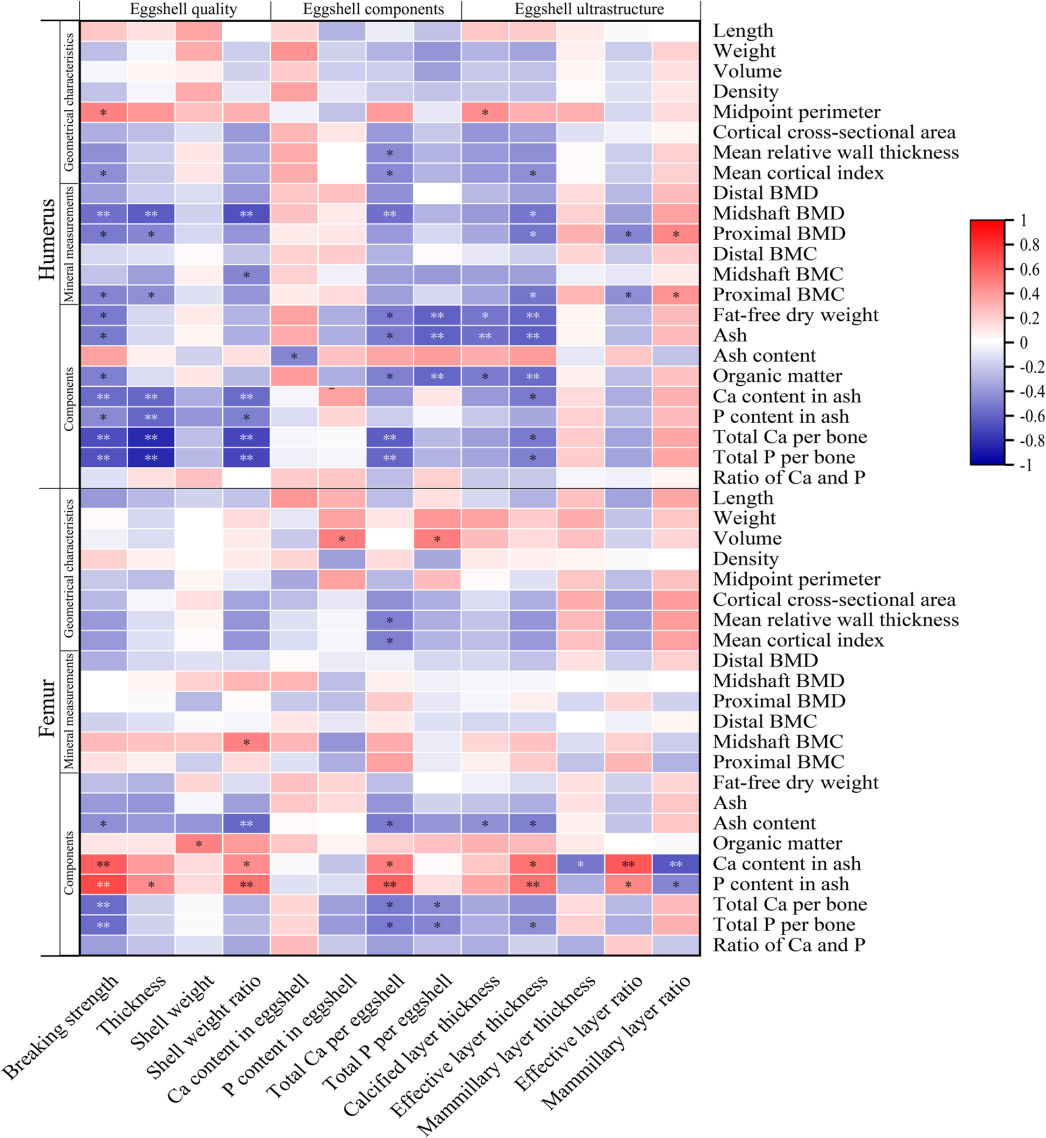
Correlation matrix for the eggshell and bone qualities. Item correlations are color graded. Red indicates positive correlations, and blue indicates negative correlations. ^*^P < 0.05, ^**^P < 0.01

Figure 4 demonstrates the correlations between eggshell and bone quality. There were negative correlations between eggshell breaking strength and mean cortical index (*r* = −0.434), midshaft BMD (*r* = −0.556), proximal BMD (*r* = −509), proximal BMC (*r* = −0.465), fat-free dry weight (*r* = −0.501), ash (*r* = −0.507), organic matter (*r* = −0.483), Ca content in ash (*r* = −0.570), P content in ash (*r* = −0.453), total Ca per bone (*r* = −0.682), total P per bone (*r* = −0.670) of the humerus as well as ash content (*r* = −0.436), total Ca per bone (*r* = −0.479) and total P per bone (*r* = −0.481) of the femur (*P* < 0.05). Eggshell thickness, weight ratio, total Ca per eggshell and effective layer thickness were negatively correlated with midshaft BMD (*r* = −0.637, −0.665, −0.555, −0.530), total Ca per bone (*r* = −0.830, −0.717, −0.623, −0.508), total P per bone (*r* = −0.837, −0.710, −0.595, −0.489) of the humerus (*P* < 0.05). Eggshell weight ratio, total Ca per eggshell, calcified layer thickness and effective layer thickness were negatively correlated with femoral ash content (*r* = −0.590, −0.508, −0.433, −0.493, *P* < 0.05). Negative correlations were also observed between: total Ca per eggshell, total P per eggshell and total Ca per bone (*r* = −0.518, −0.458), total P per bone (*r* = −0.511, −0.467) of the femurs (*P* < 0.05).

### Eggshell ultrastructure at the initiation (8.5 h PO) and growth (18.5 h PO) stages of eggshell calcification

**Fig. 5:**
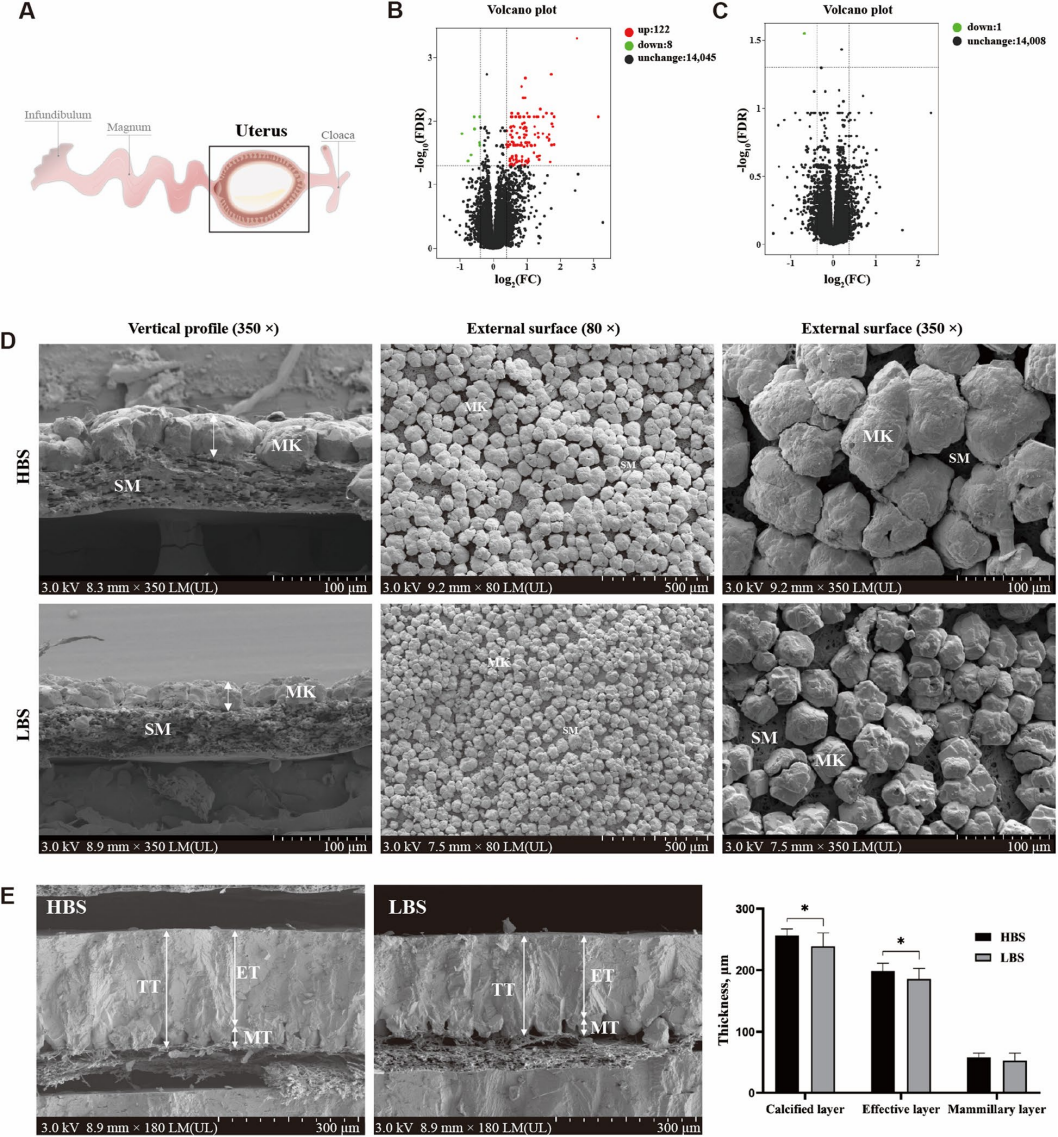
Volcano plot of DEGs and eggshell ultrastructure at the initiation (8.5 h PO) and growth (18.5 h PO) stages of eggshell calcification. **A** shows a mimetic diagram of sampling, the uterus and eggshell were removed to determine the transcriptome and ultrastructure, respectively. **B** and **D** at the initiation stage of eggshell calcification; **C** and **E** at the growth stage of eggshell calcification. SM, shell membrane; MK, mammillary knob; TT, total thickness; ET, effective layer thickness; MT, mammillary layer thickness; PO, post-oviposition; HBS, high eggshell breaking strength group; LBS, low eggshell breaking strength group. Asterisk (*) denotes significant difference (*P* < 0.05) between the HBS and LBS

As shown in Fig. 5D, at 8.5 h PO, images of vertical profiles identified the mammillary knobs were shorter in the LBS than in the HBS, indicating they grew more slowly in the LBS. Meanwhile, external ultrastructural analyses displayed the mammillary knobs were smaller and had less fusion in the LBS compared with the HBS. Additionally, at 18.5 h PO, the thickness of calcified layer and effective layer was thinner in the LBS compared to the HBS (*P* < 0.05). However, no significant difference was observed in the thickness of mammillary layer (*P* > 0.05).

### Differentially expressed genes (DEGs) in uterus at initiation (8.5 h PO) and growth (18.5 h PO) stages of eggshell calcification

A total of 48 uterine samples were obtained in the current study, of which two samples from the LBS at 8.5 h PO were rejected for further analysis, since they did not pass quality inspection. A total of 2,309,035,290 clean reads were sequenced from 46 RNA libraries, over 38.6 million reads for each sample (Additional file 3). Of these reads, 86.77%–89.66% were mapped to a unique position on the reference genome (Additional file 3). The Q30 (%) of all test samples were above 93.66%, and GC contents were stable at 48.43%–50.27%, indicating the sequence results were accurate and reliable. A total of 130 DEGs were identified at 8.5 h PO between the HBS and LBS (Fig. 5B and Additional file 4), in which 122 genes were upregulated and 8 genes were downregulated in the LBS relative to the HBS. However, volcano plot showed only 1 DEG at 18.5 h PO between the two groups (Fig. 5C). Twelve genes of each calcification stage were selected for qPCR validation, and the gene expression patterns were in accordance with the transcriptome results (Additional file 5).

### Functional enrichment analysis and gene network construction of DEGs at the initiation stage of eggshell calcification (8.5 h PO)

**Fig. 6:**
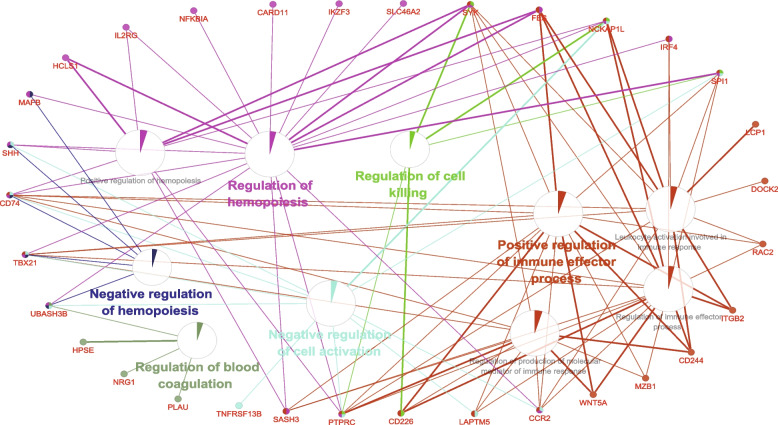
Functional enrichment analysis and gene network construction of DEGs at the initiation stage of eggshell calcification (8.5 h post-oviposition). Enrichment of DEGs that are active in regulation of cell killing, regulation of hemopoiesis, negative regulation of hemopoiesis, regulation of blood coagulation, negative regulation of cell activation, as well as positive regulation of immune effector process was noted. GO terms are presented as nodes in functionally grouped networks based on the GO cluster algorithm, where only the most significant term per group is labeled. The pie chart illustrates the percentage distribution of genes shown in the related functional groups

Functional enrichment analysis and gene network construction of DEGs at 8.5 h PO (Fig. 6) revealed the strong representation of regulation of cell killing, regulation of hemopoiesis, negative regulation of hemopoiesis, regulation of blood coagulation, as well as negative regulation of cell activation. Enrichment of DEGs that are active in positive regulation of immune effector process was also obvious. Twelve upregulated DEGs (*BCL2L14*, *C1QA*, *C1QB*, *CARD11*, *CD3E*, *HCLS1*, *IL2RB*, *NCKAP1L*, *NFKBIA*, *PRKCB*, *SYK*, *TNFAIP8L1*) were related to apoptosis and further annotated in the Additional file 6, which may suggest an active apoptosis in the LBS. Seven upregulated DEGs (*EDN2*, *IKZF1*, *PRKCB*, *UBASH3B*, *LCP1*, *MGP*, *SLC24A4*, annotated in the Additional file 6) were associated with Ca transport. Of these, *EDN2*, *IKZF1*, *PRKCB* and *UBASH3B* involved the regulation of cellular Ca ion homeostasis.

### Identification of apoptosis-related indicators in the uterus

**Fig. 7:**
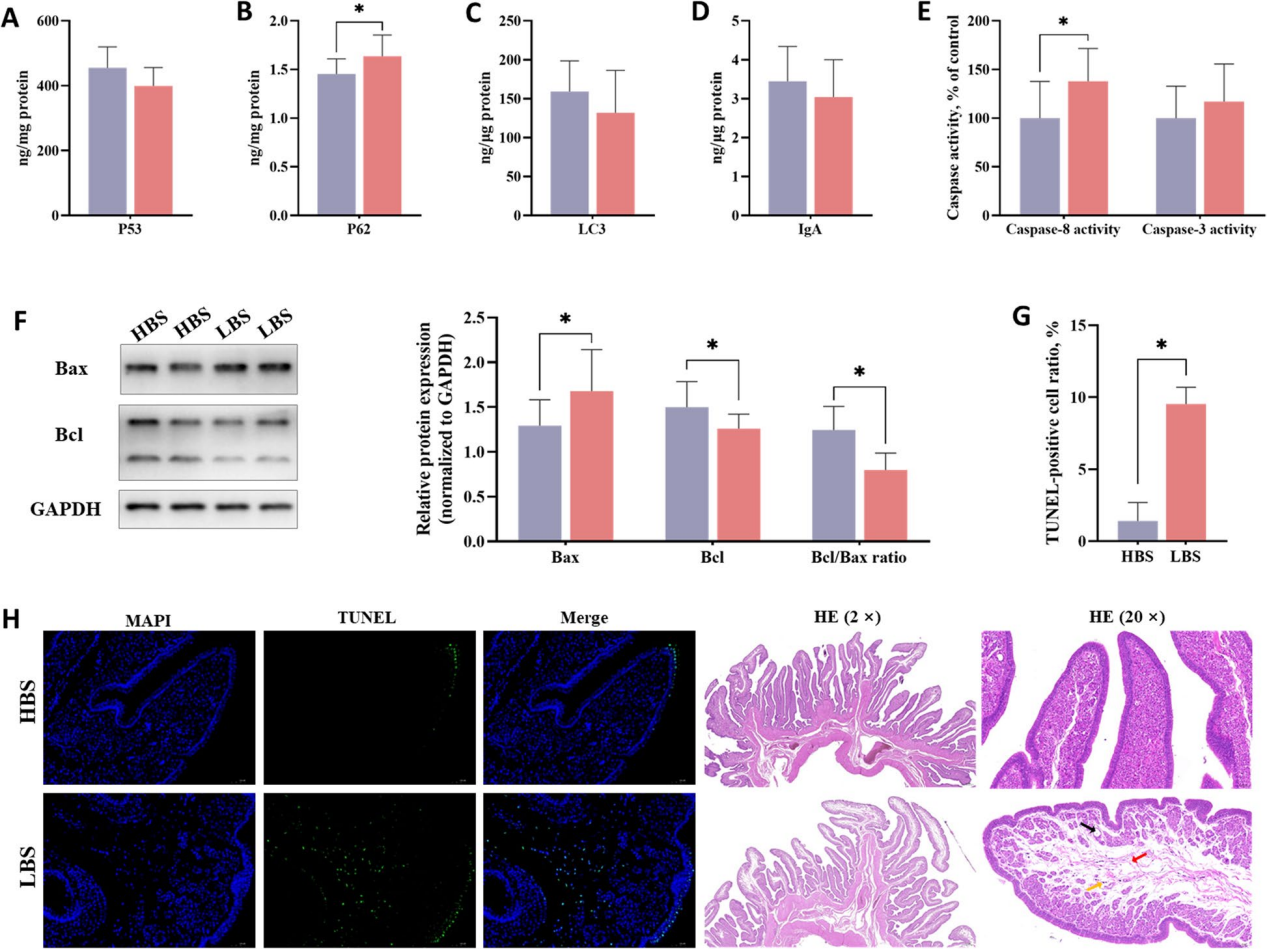
Identification of apoptosis-related indicators at the initiation stage of eggshell calcification (8.5 h post-oviposition). The protein contents of p53 (**A**), p62 (**B**), LC3 (**C**), immunoglobulin A (IgA, **D**); the activity of caspase-8 and caspase-3 (**E**); Western blot results and quantification of Bax and Bcl-2 (**F**); TUNEL-positive cells ratio (**G**); TUNEL staining (20 ×) and HE staining (**H**), interstitial edemas (black arrow), loose connective tissue (red arrow) and inflammatory cell infiltration (yellow arrow). HBS, high eggshell breaking strength group; LBS, low eggshell breaking strength group. Asterisk (*) denotes significant difference (*P* < 0.05) between the HBS and LBS

As shown in Fig. 7, the level of p62 protein was significantly higher in the LBS (*P* < 0.05). The activity of caspase-8 was increased in the LBS (*P* < 0.05). The LBS significantly increased the relative protein expression of Bax, while reducing Bcl-2 expression and the ratio of Bcl-2/Bax (*P* < 0.05). Representative images of TUNEL assay are illustrated in Fig. 7H. The uterus of the LBS showed a significant increase in TUNEL-positive cells (*P* < 0.05, Fig. 7G). The HE staining (Fig. 7H) showed that the uterine tissue from both groups had an intact epithelial structure, with the epithelial cells arranging tightly. However, the uterine tissue in the LBS exhibited multiple interstitial edemas and loose connective tissue with a few inflammatory cell infiltrations.

### Quantification of bone remodeling-related mRNA in bones at initiation (8.5 h PO) and growth (18.5 h PO) stages of eggshell calcification

**Fig. 8:**
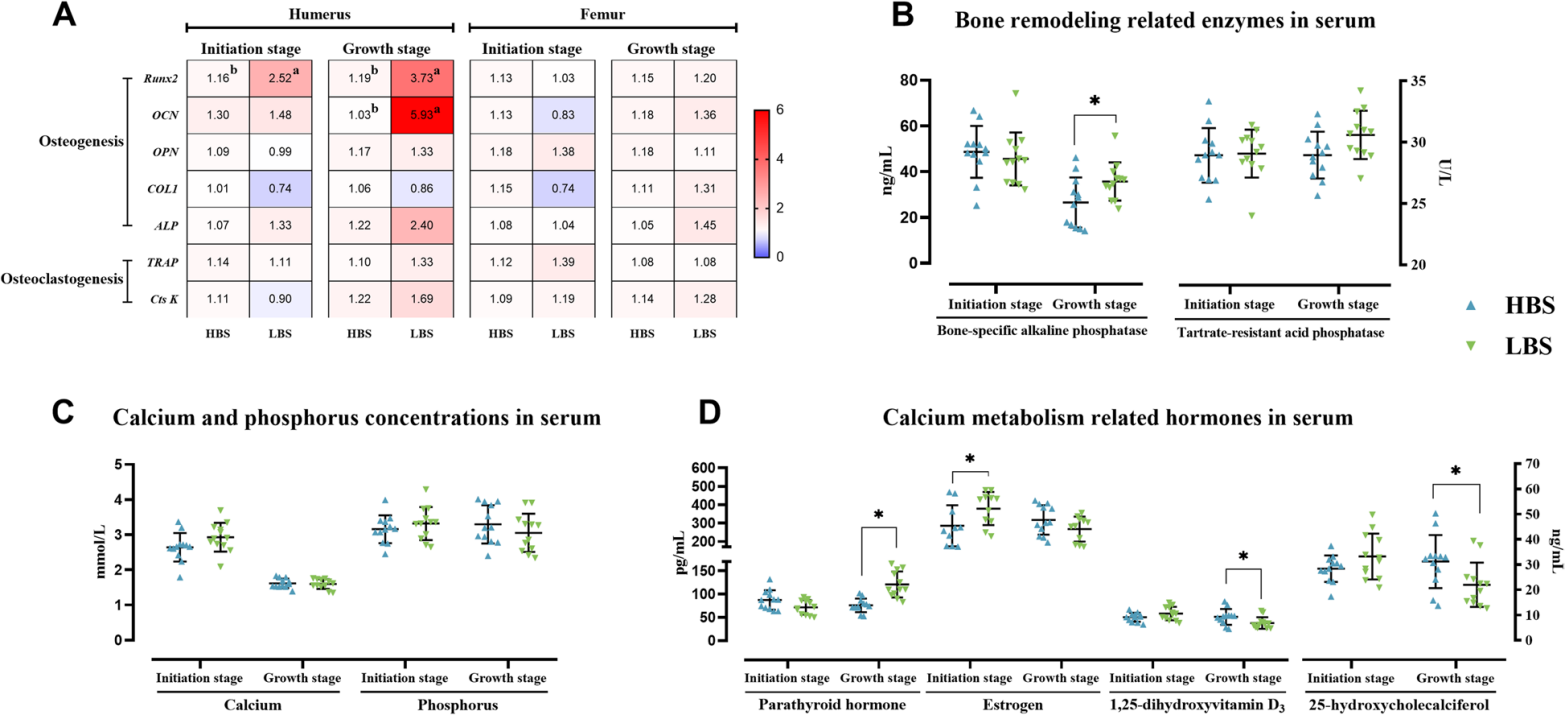
Differences in the bone remodeling markers and hormones of the hens laying eggs with different eggshell breaking strength. The quantification of bone remodeling related mRNA in bone (**A**), bone remodeling related enzymes (**B**) as well as calcium and phosphorus concentrations (**C**) and calcium metabolism related hormones (**D**) in serum. *Runx2*, runt-related transcription factor 2; *OCN*, osteocalcin; *OPN*, osteopontin; *COL1*, collagen 1; *ALP*, alkaline phosphatase; *TRAP*, tartrate-resistant acid phosphatase; *Cts K*, cathepsin K. HBS, high eggshell breaking strength group; LBS, low eggshell breaking strength group. Different superscripts in the adjacent cells indicate significant differences (*P* < 0.05). Asterisk (*) denotes significant difference (*P* < 0.05) between the HBS and LBS

Figure 8A demonstrates the relative expression levels of bone remodeling genes. At 8.5 h PO, a significant increase of runt-related transcription factor 2 (*Runx2*) expression was shown in the humerus of the LBS compared to that of the HBS (*P* < 0.05), while no significant differences were observed in the expression levels of other genes (*P* > 0.05). The expressions of humeral *Runx2* and *OCN* genes were significantly upregulated in the LBS compared with those in the HBS (*P* < 0.05). No significant differences were observed in the expression of bone remodeling-related genes in femurs (*Runx2*, *OCN*, *OPN*, *COL1*, *ALP*, *TRAP*, *Cts K*, *P* > 0.05).

### Changes of calcium, phosphorus, bone remodeling-related enzymes and hormones in serum during eggshell calcification

The activity of TRAP and the levels of BALP, Ca, P, PTH, E_2_, 1,25(OH)_2_D_3_ and 25(OH)D_3_ are presented in Fig. 8B–D. Compared with the HBS, the LBS had a higher serum BALP level at 18.5 h PO (*P* < 0.05) while no difference was observed at 8.5 h PO (*P* > 0.05). No significant differences appeared in the activity of TRAP, nor in the levels of Ca and P between the HBS and LBS at both calcification periods (*P* > 0.05). The LBS significantly increased the level of serum PTH, but decreased that of 1,25(OH)_2_D_3_ and 25(OH)D_3_ at 18.5 h PO (*P* < 0.05). At 8.5 h PO, the serum E_2_ level was significantly higher in the LBS than in the HBS (*P* < 0.05), while there were no differences in the levels of PTH, 1,25 (OH)_2_D_3_ and 25(OH)D_3_ (*P* > 0.05).

## Discussion

Eggshell breaking strength is an important egg quality trait. In the current study, the selected hens were from a flock with an average breaking strength of 34.55 N, and 20% of the hens in this flock laying eggs with eggshell breaking strength lower than 29 N. The eggs laid by these hens may be more prone to break and crack during the collection and transportation [3]. Consistent with a previous study [39], the eggs with low eggshell breaking strength were accompanied by a thinner thickness, which was also reflected by an ultrastructural observation that LBS group showed a thinner effective layer and subsequently total calcified layer. The eggshell effective layer comprises about two thirds of the total calcified layer and plays crucial roles in resisting the inception and propagation of cracks [40]. The formation of eggshell ultrastructure is largely affected by the deposition of calcium carbonate during its calcification process [41]. As predicted, the eggshell of the LBS showed a lower weight, weight ratio and total Ca content. Taken together, eggshell breaking strength reduction in late laying period may be related to the deterioration of ultrastructure (decreased effective layer) caused by the decrease of calcium carbonate deposition.

The correlation between eggshell and bone quality may be different in various conditions since eggshell and bone quality were also influenced by diets and environmental factors [6–9, 31, 42]. Our previous study suggested that eggshell quality exhibited simultaneous improvements with bone quality by changing a conventional caging system to an aviary system [31]. However, the present study exhibited negative correlations between bone and eggshell quality under the same feed and environmental factors, indicating that the HBS had higher risks for osteoporosis. This is in accordance with previous studies that reported hens with hard-shelled eggs or high production had poor-quality skeletons [3, 30]. The negative correlations between eggshell and bone quality were mainly reflected in the components. It suggested that the bone remodeling tended to preserve bone mass in the hens with weak-shelled eggs, while it was prone to lose bone mass to address the Ca needs of eggshell calcification in the hens with hard-shelled eggs. Thus, under the same diet and environment, the negative correlations between eggshell and bone quality may be associated with the competition and delivery of Ca between the uterus and bones.

The uterus transports Ca to meet the Ca demands of eggshell calcium carbonate deposition at different stages of eggshell calcification. During the initiation stage of eggshell calcification, calcite crystals radially grow around the mammillary cones, then the adjacent mammillary knobs gradually come together and fuse at the spatial competition, forming the bases of palisade layer [43]. During this period, eggshell mammillary knobs grew slowly with few fusions in the LBS, indicating a decreased deposition of calcium carbonate. However, such difference did not affect the frequency of abnormal mammillary knobs (Additional file 7) in the ultrastructural characterization of intact eggshells. During the growth stage of eggshell calcification, the eggshell effective layer thickness was thinner in the LBS, which was attributed to less early fusion of mammillary knobs at the initiation stage of eggshell calcification as well as reduced calcium carbonate deposition at the growth stage of eggshell calcification. Thus, the eggshell ultrastructural deterioration (decreased effective layer thickness) was related to the decreasing of calcium carbonate deposition at both initiation and growth stages of eggshell calcification.

Uterus transports Ca and secretes matrix proteins to regulate the deposition of calcium carbonate during eggshell calcification. The uterine transcriptome analysis showed that the differences of calcium carbonate deposition were mainly caused by the transcriptional variations at the initiation stage of eggshell calcification, since only a few DEGs were observed at the growth stage, while 130 genes were differentially expressed at the initiation stage. The mass transport of Ca in the uterus is required for the maintenance of eggshell calcification. In this study, 7 DEGs (*EDN2*, *IKZF1*, *PRKCB*, *UBASH3B*, *LCP1*, *MGP*, and *SLC24A4*) were associated with Ca transport, of which *EDN2*, *IKZF1*, *PRKCB* and *UBASH3B* involved cellular Ca ion homeostasis and positive regulation of cytosolic Ca ion concentration. Their upregulations in the LBS may induce a rise in cytosolic Ca ion concentration, which could lead to apoptosis due to the cellular Ca overload [44, 45]. In agreement with this, the upregulation of a GO cluster relevant for cell killing along with the genes related to apoptosis (*BCL2L14*, *C1QA*, *C1QB*, *CARD11*, *CD3E*, *CSF1R*, *E2F8*, *HCLS1*, *IL2RB*, *NCKAP1L*, *NFKBIA*, *PRKCB*, *SPI1*, *SYK*, and *TNFAIP8L1*) suggested an excessive apoptosis, which may aggravate tissue damages in the LBS. MGP is a negative regulator for vascular calcification [46], and its upregulation of gene expression may lead to a disorder in chickens via inhibiting Ca-dependent function [47]. LCP1 is of great significance in the adipogenesis and lipid metabolism, and the overexpression of *LCP1* could suppress lipid catabolism and increase adipogenesis and lipogenesis [48]. The lipid accumulation in the aged laying hens would hinder uterine function [49] and interfere eggshell calcification [50]. The SLC24A4 family exports Ca out of the cell with the potassium via entry of sodium [51, 52]. However, its localization in the uterus and biological function in eggshell calcification were not clear and require further inquiry. Additionally, the enrichment of a GO cluster on hemopoiesis linked with tissue repair may contribute to the repair of tissue damage that result from apoptosis-mediated cell death [53]. The upregulation of *NRG1* was concordant with this, which is involved in the development and regeneration of the chicken reproductive tract through mediating E_2_ [54]. The differences in the immune repones between the hens with different eggshell breaking strength, such as the positive regulation of immune effector process and regulation of blood coagulation, could be due to the phagocytic clearance of apoptotic cells. Overall, the hens with weak-shelled eggs may induce intracellular Ca overload to trigger excessive apoptosis and aggravate uterine tissue damages, and hematopoietic repair may be involved in the subsequent repair of injury.

Maintenance of tissue integrity is fundamental to transport Ca in the uterus. Although, as a compensatory mechanism, the hematopoiesis system was activated and exerted its repair property, the extents of uterine injury and repair were not understood. Thus, the apoptosis and tissue homeostasis indexes were further analyzed in the current study to assess the mechanism underlying the decrease in the eggshell Ca deposition. Autophagy, an evolutionarily conserved process among eukaryotes, plays a critical role in homeostasis in cells and tissues by the clearance of detrimental and damaged proteins and dysfunctional organelles [55]. The levels of p62 and LC3 identify the progression of autophagy induction, of which the former is an autophagy substrate, and the latter increases synchronously with increased autophagic flux [56]. An increased level of p62 with a constant LC3 in the LBS hinted a disruption in the downstream steps of autophagy, that was unable to clear autophagosomes and degrade p62 [57]. On the one hand, this could lead to the cellular dysfunction and aggravate apoptosis [58]. On the other hand, autophagy defect may facilitate excessive inflammatory responses and create tissue damages [59]. Apoptosis acts as an important defense mechanism of host against infection, in which caspase 8, caspase 3, Bcl-2 and Bax play key roles. The increased caspase 8 activity in the LBS indicates the initiation of extrinsic apoptotic pathway, which could enhance the clearance of virus-infected cells [60]. Bcl-2 (anti-apoptotic) and Bax (pro-apoptotic) belong to Bcl-2 family that is a central regulator of apoptosis, the reduction in Bcl-2/Bax ratio of the LBS suggested cell death in the apoptosis response [61]. Activation of the pro-apoptotic effect in the LBS was further confirmed by an increase of TUNEL-positive cells in its uterus. Additionally, most apoptotic cells were localized at the inner of the uterine fold, which was consistent with the HE staining result that revealed the edema or dissolution in tubular glands. In general, the uterus of the LBS was more sensitive to apoptosis and tissue damages at the initiation stage of eggshell calcification. Similarly, HE staining showed the LBS also had obvious tissue damage at the growth stage of eggshell calcification. Uterine tissue injury is bound to hamper Ca transport, reducing eggshell quality even inducing weak-shelled and soft-shelled eggs [62, 63]. Thus, the breaking strength reduction and ultrastructural deterioration of eggshells may be attributed to a declined Ca transport due to uterine tissue damages.

The blockage of Ca transport resulting from tissue damage may influence the deposition and release of skeletal Ca. Although the expression levels of genes related to bone remodeling did not differ significantly in the femur, the geometrical and compositional changes suggested increased bone resorption in the femur of HBS. In the current study, femoral geometric metrics showed no difference in the midpoint perimeter, while significant decreases in the mean relative wall thickness and mean cortical index were observed in the HBS, leading to a larger endocortical bone diameter. Such enlarged diameter and more structural damages of endocortical bones, such as irregular erosions in the endocortical surface and more demineralized regions in the intracortical region, are usually representative of endocortical resorption in human beings [64, 65]. Therefore, more resorption could occur in the femur of hens laid hard-shelled eggs to meet the Ca requirement during eggshell calcification, in return decreased bone quality.

Humerus exhibited similar variations as the femur manifested by enlarged endocortical diameter and the lower skeletal minerals and components in the HBS. However, unlike the femur, such difference in the humerus may be related to more endocortical formation in the LBS rather than more endocortical resorption in the HBS, since the LBS had a thicker layer of woven bone that lined the intracortical portion of the bone. The woven bone, an intermediate form of bone development, represented the initiation of intramembranous ossification in the LBS [66]. *Runx2* is involved in the regulation of genes responsible for the biosynthesis of bone-specific protein [66, 67]. At the growth stage of eggshell calcification, increased *Runx2* in the LBS appeared to upregulate *OCN* expression that would facilitate the biosynthesis of skeletal organic matrix [68] and the anchoring of Ca and phosphate [19], which is a prerequisite for the increment of the cortical bone formation. Additionally, BALP is a typical serum marker that reflects the rate of bone formation in bone tissue [69], and increased serum BALP level in the LBS indicated a more intense osteogenesis in bones. Thus, the humerus of hens with weak shells had more bone formation at the growth stage of eggshell calcification. The coupling of bone formation and bone resorption maintain bone homeostasis. During eggshell formation, following the bone resorption, bone displays an intense osteoblastic activity that remodels new bone for the next cycle of eggshell calcification [70]. The uterus of the LBS did not require extensive Ca consumption due to its blockage of Ca transport, thus Ca redundancy obtained by bone resorption may be recovered to perform new bone formation. In contrast, in the HBS, bone formation may be inhibited in the humerus due to an increased acquisition of Ca in the uterus.

Blood Ca concentration and its regulatory factors may be the key signaling for the crosstalk between uterus and skeletons due to their highly dependence on the Ca. PTH and 1,25(OH)_2_D_3_ are two major calcium-regulating hormones that involved in Ca homeostasis during eggshell calcification by directly and indirectly mediating bone remodeling and intestinal Ca absorption [71]. The effect of PTH on bones is contingent on the periodicity of the PTH signal [72], and the intermittent increase of the serum PTH level would result in an anabolic effect on rat metatarsal [73]. Thus, increased humerus formation in the LBS may be related to the temporary increase of serum PTH at the growth stage of eggshell calcification. Increased serum PTH may account for the enhancement of Runx2-dependent transcription via mediating Runx2 protein expression in the LBS [74], thereby stimulating the synthesis of bone biomarkers associated with formation. The secretion of PTH is subject to a direct regulation of calcium-sensing receptors and a negative feedback regulation by vitamin D receptors [75, 76]. In the LBS, the diminished serum active vitamin D_3_ may maintain the PTH at a high level by attenuating negative regulation. Inflammation and immune response evoked with uterine damages may be a possible reason for the reduction in the circulating levels of 25(OH)D_3_ and 1,25(OH)_2_D_3_, since inflammation could affect vitamin D_3_ metabolism by mediating the downregulation of 1α-hydroxylase and the upregulation 24-hydroxylase [77]. Thus, the uterine damage may affect bone remodeling through the regulation of vitamin D_3_ metabolism and the PTH level. This study provides a possible pathway associated with hormones for the signal transmission between bone and uterus.

## Conclusions

In conclusion, in aged laying hens, the lower eggshell breaking strength may be attributed to a declined Ca transport due to uterine tissue damages, which could affect eggshell calcification and lead to a weak ultrastructure. Impaired Ca transport in the uterus may result in reduced femoral bone resorption and increased humeral bone formation to maintain a higher minerals and bone quality in the LBS. Blood hormones such as PTH, 1,25(OH)_2_D_3_ and 25(OH)D_3_ may be acting as mediators involved in signaling between bone and uterus.

### Supplementary Information

**Additional file 1: Table S1. **Ingredient and nutrient levels of the experimental diets (air-dried basis).

**Additional file 2: Table S2. **The primers used for qRT-PCR assays.

**Additional file 3: Table S3.** Alignment of the samples’ sequence with the reference genome (*Gallus gallus GRCg6*).

**Additional file 4.** Differentially expressed genes (DEGs) in uterus at initiation (8.5 h PO) and growth (18.5 h PO) stages of eggshell calcification.

**Additional file 5: Fig. S1.** Quantitative PCR (qPCR) validation of RNA sequencing (RNA-Seq) results. **a** The initiation stage of eggshell calcification; **b** The growth stage of eggshell calcification. Asterisks (*) denotes significance (FDR < 0.05 in the RNA-seq, *P* < 0.05 in the qPCR). CD3E, CD3e molecule; ITGB2, integrin subunit beta 2; CARD11, caspase recruitment domain family member 11; MZB1, marginal zone B and B1 cell specific protein; NCKAP1L, NCK associated protein 1 like; NRG1, neuregulin 1; PTPRC, protein tyrosine phosphatase, receptor type C; SPI1, Spi-1 proto-oncogene; SYK, spleen associated tyrosine kinase; BCL2L14, BCL2 like 14; CA2, carbonic anhydrase 2; CALB1, calbindin 1; CAPN6, calpain 6; ATP2B1, ATPase plasma membrane Ca^2+^ transporting 1; ATP2B2, ATPase plasma membrane Ca^2+^ transporting 2; ITPR1, inositol 1,4,5-trisphosphate receptor type 1; SLC8A1, solute carrier family 8 member A1; SLC8A3, solute carrier family 8 member A3; SLC4A4, solute carrier family 4 member 4; SLC26A9, solute carrier family 26 member 9; CLCN5, chloride voltage-gated channel 5; ATP2A2, ATPase sarcoplasmic/endoplasmic reticulum Ca^2+^ transporting 2.

**Additional file 6: Table S4.** Analysis of differentially expressed genes (|fold change| > 1.3 at a false discovery rate < 0.5) in association with apoptosis and calcium transport.

**Additional file 7: Table S5.** Comparison of ultrastructural variations in eggshell mammillary layer of the hens laying eggs with different eggshell breaking strength.

## Data Availability

RNA-sequencing datasets are available in the National Center for Biotechnology Information (accession numbers: PRJNA1000560).

## Abbreviations

1,25(OH)_2_D_3_: 1,25-dihydroxyvitamin D_3_
25(OH)D_3_: 25-hydroxyvitamin D_3_
ALP: Alkaline phosphatase
BALP: Bone-specific alkaline phosphatase
BMC: Bone mineral content
BMD: Bone mineral density
COL1: Collagen type I
Cts K: Cathepsin K
DEGs: Differentially expressed genes
E_2_: Estrogen
GO: Gene Ontology
HBS: High eggshell breaking strength group
HE: Hematoxylin-eosin
LBS: Low eggshell breaking strength group
OCN: Osteocalcin
OPN: Osteopontin
PO: Post-oviposition
PTH: Parathyroid hormone
Runx2: Runt-related transcription factor 2
SD: Standard deviation
TRAP: Tartrate resistant acid phosphatase
