# Supplementary material for: Decreased eggshell strength caused by impairment of uterine calcium transport coincide with higher bone minerals and quality in aged laying hens
Source: J Anim Sci Biotechnol. 2024 Mar 4;15:37. doi: 10.1186/s40104-023-00986-2 (PMC10910863; doi:10.1186/s40104-023-00986-2)
Supplement: Supplementary file 1 — Additional file 1: Table S1. Ingredient and nutrient levels of the experimental diets (air-dried basis). [file 40104_2023_986_MOESM1_ESM.docx]

**Additional file 1**

**Table S1** Ingredient and nutrient levels of the experimental diets (air-dried basis)

| **Ingredient, %** | **Content** | **Nutrient level^2^, %** | | **Content** |
| --- | --- | --- | --- | --- |
| Corn | 59.00 | AME, MJ/kg | 11.11 | |
| Soybean meal | 24.53 | Crude protein | 16.37 (16.41) | |
| Soybean oil | 1.80 | Calcium^3^ | 3.99 (3.89) | |
| Limestone | 10.60 | Methionine | 0.37 | |
| DL-Methionine | 0.12 | Lysine | 0.80 | |
| 50% choline chloride | 0.12 | Total phosphorus^3^ | 0.46 (0.43) | |
| Calcium hydrogen phosphate | 0.90 | Available phosphorus | 0.26 | |
| Sodium chloride | 0.15 | Methionine + Cysteine | 0.65 | |
| Sodium sulfate | 0.20 | Ratio of calcium and total phosphorus | 8.67:1  (9.05:1) | |
| Wheat bran | 2.40 |  |  |  |
| Vitamin and mineral premix^1^ | 0.18 |  |  | |
| Total | 100.00 |  |  | |

^1^ Premix provided the following per kg of the diet: vitamin A, 9,500 IU; vitamin D_3_, 4,125 IU; vitamin E, 15 IU; vitamin K, 2 mg; thiamine, 1 mg; riboflavin, 8.5 mg; calcium pantothenate, 11 mg; niacin, 32.5 mg; pyridoxine, 8 mg; biotin, 0.5 mg; folic acid, 1.25 mg; vitamin B_12_, 0.02 mg; Mn, 65 mg; I, 1 mg; Fe, 60 mg; Cu, 8 mg; Zn, 66 mg; phytase, 500 mg

^2^ Nutrient levels are calculated values

^3^ Numbers in parentheses are the analyzed value
