# Supplementary material for: Decreased eggshell strength caused by impairment of uterine calcium transport coincide with higher bone minerals and quality in aged laying hens
Source: J Anim Sci Biotechnol. 2024 Mar 4;15:37. doi: 10.1186/s40104-023-00986-2 (PMC10910863; doi:10.1186/s40104-023-00986-2)
Supplement: Supplementary file 2 — Additional file 2: Table S2. The primers used for qRT-PCR assays. [file 40104_2023_986_MOESM2_ESM.docx]

**Additional file 2**

**Table S2** The primers used for qRT-PCR assays

| **Gene name** | **Primer sequence (5′→3′)** | **Accession** | **Efficiency, 100%** |
| --- | --- | --- | --- |
| *β-actin* | F: TATGTGCAAGGCCGGTTTC  R: TGTCTTTCTGGCCCATACCAA | NM_205518.2 | 97.22 |
| *CD3E* | F: CTGCGGATCTTCTCATCACCTTGG | NM_001397708.1 | 98.64 |
|  | R: GCTCGTAGTCTGGGTTTGGAACAG |  |  |
| *ITGB2* | F: GCCTTCAAGCACATCCTCTCAC | NM_205251.2 | 109.55 |
|  | R: CCAGCCAATCAAGTCTCCACAC |  |  |
| *CARD11* | F: AGAGGTGCCACGGTCCAGTAAC | NM_001006161.4 | 106.14 |
|  | R: GGAGATGTTCAGATTCAGGCGGATG |  |  |
| *MZB1* | F: CTTGCAGGACTGGGAGCTTTACG | XM_003642067.5 | 94.55 |
|  | R: CGAGGCTGTGGCACATCTTGG |  |  |
| *NCKAP1L* | F: CCTGCCTGCTGCTGGTCTTTG | NM_001031339.3 | 98.25 |
|  | R: GTAGCCATCCATCTCGGTGTTGTAG |  |  |
| *NRG1* | F: CACCTTCAGCAATTCAACTCCTTCC | NM_204117.2 | 92.54 |
|  | R: GCGTGGTCTCATACTCTTCATCCTC |  |  |
| *PTPRC* | F: GCAGGAGTTCAGCCAGCATCAAC | NM_204417.3 | 106.04 |
|  | R: AGTGAGAGTGCGTGTGGTGAGG |  |  |
| *SPI1* | F: CCTATTTGCCTCGGATGTGCCTAC | NM_001389369.2 | 97.70 |
|  | R: TCTGCCTCTCAATGTCCTCCTCATC |  |  |
| *SYK* | F: CAACCCTGCTAACCATTTGCCATAC | NM_001031430.2 | 103.98 |
|  | R: GTCCATCGCTCATTCCTCCTTGC |  |  |
| *BCL2L14* | F: CCACTTCTGACACCTGCACTGAG | XM_004938051.5 | 108.66 |
|  | R: GAGATGGCTGAGACCTGGTAGTAAC |  |  |
| *CA2* | F: CTCCTCCGACAAGTCAGTGC | NM_205317.1 | 93.14 |
|  | R: TACGACGGCCAAACCATCAG |  |  |
| *CALB1* | F: TGTTATGGAGTGCAGGATGG | NM_205513.1 | 99.32 |
|  | R: TAGAGCGAACAAGCAGGTGA |  |  |
| *CAPN6* | F: TGTCTTCGTCGGTTCAACTCTTCAG | NM_206904.2 | 91.39 |
|  | R: GCTGTCCCTGCTGGATACATTGC |  |  |
| *ATP2B1* | F: CTGCACTGAAGAAAGCAGATGTTG | XM_416133.2 | 107.20 |
|  | R: GCTGTCATATACGTTTCGTCCCC |  |  |
| *ATP2B2* | F: TTACTGTACTTGTGGTTGCTGTCCC | XM_001231767.1 | 104.07 |
|  | R: GGTTGTTAGCGTCCCTGTTTTG |  |  |
| *ITPR1* | F: TGAATGCCACCAGCGACAGAG | NM_001174059.2 | 105.69 |
|  | R: TGTAGTGTTGCCTCCTTCCAGAAG |  |  |
| *SLC8A1* | F: GGATTGTGGAGGTTTGGGAAGG | NM_001398209.1 | 108.19 |
|  | R: CTGTTTGCCAGCTCGGTATTTC |  |  |
| *SLC8A3* | F: GGAGAGACCACAACAACAACCATTC | NM_001293097.3 | 107.27 |
|  | R: AGCTACGAATCCATGCCCACAC |  |  |
| *SLC4A4* | F: GAAGGCGGCGTCATAGGAGAAG | XM_420603.8 | 101.37 |
|  | R: GTTTGAGGATGCTGCTGCTTGTC |  |  |
| *SLC26A9* | F: GCCTCTTCGATGAGGAGTTTGAG | XM_015299071.4 | 101.30 |
|  | R: CTGACCCCACCAAGAACATCAG |  |  |
| *CLCN5* | F: ATTGGGCGGGAGGGCATTTAC | XM_040670258.2 | 91.08 |
|  | R: TGTGAGAACTCTTCCTTGGCTTCC |  |  |
| *ATP2A2* | F: GCAGCTTGCATATCTTTTGTGCTG | NM_001271974.2 | 109.70 |
|  | R: CATTTCTTTCCTGCCACACTCC |  |  |
| *Runx2* | F: CACGCTGCTAAACCCAAACT  R: GACTCATCCATCCTGCCACT | NM_204128.2 | 91.91 |
| *OCN* | F: GAAGAGGCAGAAGAGGTTCG  R: AGATAGTCACAGGGAGGGTAGC | NM_001201386.2 | 107.86 |
| *OPN* | F: TAGGAGTTGCTGCTGGGATT | NM_204535.5 | 102.12 |
|  | R: CCTGGTGGTACCTGTGTGTG |  |  |
| *COL1* | F: TTGACCCTAACCAAGGATGC | NM_001079714.2 | 104.86 |
|  | R: CACCCCTTCTGCGTTGTATT |  |  |
| *ALP* | F: GGAGAAGGACCCCGAATACTG  R: TTGACGCCGCAGAGGTAAG | NM_205360.2 | 101.83 |
| *TRAP* | F: CTGGCTTTGGGCGATAACT  R: TCGGAGTGTCGGCTGTATG | XM_040693093.2 | 92.71 |
| *Cts K* | F: ACGTCCCGGAGGTTGATTTG | NM_204971.3 | 95.16 |
|  | R: CCACCTCCTCGCTGGTCATA |  |  |

F forward primer, R reverse primer

*CD3E*, CD3e molecule; *ITGB2*, integrin subunit beta 2; *CARD11*, caspase recruitment domain family member 11; *MZB1*, marginal zone B and B1 cell specific protein; *NCKAP1L*, NCK associated protein 1 like; *NRG1*, neuregulin 1; *PTPRC*, protein tyrosine phosphatase, receptor type C; *SPI1*, Spi-1 proto-oncogene; *SYK*, spleen associated tyrosine kinase; *BCL2L14*, BCL2 like 14; *CA2*, carbonic anhydrase 2; *CALB1*, calbindin 1; *CAPN6*, calpain 6; *ATP2B1*, ATPase plasma membrane Ca^2+^ transporting 1; *ATP2B2*, ATPase plasma membrane Ca^2+^ transporting 2; *ITPR1*, inositol 1,4,5-trisphosphate receptor type 1; *SLC8A1*, solute carrier family 8 member A1; *SLC8A3*, solute carrier family 8 member A3; *SLC4A4*, solute carrier family 4 member 4; *SLC26A9*, solute carrier family 26 member 9; *CLCN5*, chloride voltage-gated channel 5; *ATP2A2*, ATPase sarcoplasmic/endoplasmic reticulum Ca^2+^ transporting 2; *Runx2*, runt-related transcription factor 2; *OCN*, osteocalcin; *OPN*, osteopontin; *COL1*, collagen 1; *ALP*, alkaline phosphatase; *TRAP*, tartrate-resistant acid phosphatase; *Cts K*, cathepsin K
