# Supplementary material for: Decreased eggshell strength caused by impairment of uterine calcium transport coincide with higher bone minerals and quality in aged laying hens
Source: J Anim Sci Biotechnol. 2024 Mar 4;15:37. doi: 10.1186/s40104-023-00986-2 (PMC10910863; doi:10.1186/s40104-023-00986-2)
Supplement: Supplementary file 3 — Additional file 3: Table S3. Alignment of the samples’ sequence with the reference genome (Gallus gallus GRCg6). [file 40104_2023_986_MOESM3_ESM.docx]

**Additional file 3**

**Table S3** Alignment of the samples’ sequence with the reference genome (*Gallus gallus GRCg6*)

| **Sample name** | **Total No. reads** | **Mapped reads** | **Percentage of mapped reads** | **Uniq mapped reads** | **Percentage of uniq mapped Reads** | **Multiple mapped reads** | **Percentage of multiple mapped reads** |
| --- | --- | --- | --- | --- | --- | --- | --- |
| H1 | 52,861,394 | 48,114,607 | 91.02% | 46,999,807 | 88.91% | 917,705 | 2.38% |
| H2 | 42,025,032 | 38,337,609 | 91.23% | 37,574,281 | 89.41% | 1,513,016 | 3.64% |
| H3 | 45,806,814 | 41,709,993 | 91.06% | 40,726,735 | 88.91% | 814,572 | 1.95% |
| H4 | 53,072,158 | 48,503,062 | 91.39% | 47,354,590 | 89.23% | 763,328 | 1.82% |
| H5 | 50,409,146 | 46,246,338 | 91.74% | 45,194,679 | 89.66% | 954,838 | 2.27% |
| H6 | 40,453,766 | 36,920,315 | 91.27% | 36,042,779 | 89.10% | 936,570 | 2.20% |
| H7 | 40,116,516 | 36,600,997 | 91.24% | 35,754,307 | 89.13% | 1,006,601 | 2.26% |
| H8 | 60,812,934 | 55,511,572 | 91.28% | 54,136,115 | 89.02% | 921,619 | 2.07% |
| H9 | 44,581,734 | 40,561,618 | 90.98% | 39,555,017 | 88.72% | 978,464 | 2.15% |
| H10 | 41,822,518 | 38,037,642 | 90.95% | 37,223,070 | 89.00% | 868,590 | 2.17% |
| H11 | 52,322,530 | 47,909,202 | 91.57% | 46,829,486 | 89.50% | 846,690 | 2.11% |
| H12 | 45,427,770 | 41,420,291 | 91.18% | 40,441,827 | 89.02% | 877,536 | 2.17% |
| L1 | 64,972,710 | 59,172,285 | 91.07% | 57,675,094 | 88.77% | 983,258 | 2.15% |
| L4 | 52,006,038 | 46,443,381 | 89.30% | 45,395,504 | 87.29% | 1,049,788 | 2.21% |
| L5 | 48,688,556 | 44,126,684 | 90.63% | 43,071,169 | 88.46% | 1,060,385 | 2.20% |
| L6 | 49,017,098 | 44,396,713 | 90.57% | 43,445,840 | 88.63% | 1,102,126 | 2.28% |
| L7 | 58,369,782 | 52,898,237 | 90.63% | 51,641,748 | 88.47% | 1,055,515 | 2.17% |
| L8 | 48,739,042 | 44,355,986 | 91.01% | 43,335,466 | 88.91% | 1,020,520 | 2.09% |
| L9 | 51,567,282 | 46,853,080 | 90.86% | 45,843,527 | 88.90% | 950,873 | 1.94% |
| L10 | 49,236,824 | 44,713,937 | 90.81% | 43,715,829 | 88.79% | 1,033,764 | 2.21% |
| L11 | 46,899,296 | 42,269,085 | 90.13% | 41,245,180 | 87.94% | 1,023,905 | 2.18% |
| L12 | 64,391,806 | 58,666,673 | 91.11% | 57,284,684 | 88.96% | 995,948 | 2.11% |
| NH1 | 48,125,042 | 43,483,667 | 90.36% | 42,423,282 | 88.15% | 998,108 | 2.03% |
| NH2 | 52,526,740 | 47,365,569 | 90.17% | 46,238,843 | 88.03% | 1,009,553 | 1.96% |
| NH3 | 53,351,776 | 48,363,415 | 90.65% | 47,180,854 | 88.43% | 1,047,877 | 2.01% |
| NH4 | 41,581,180 | 37,594,162 | 90.41% | 36,081,146 | 86.77% | 1,225,062 | 2.34% |
| NH5 | 42,541,704 | 38,468,939 | 90.43% | 37,532,369 | 88.22% | 1,079,716 | 2.06% |
| NH6 | 49,659,514 | 44,998,339 | 90.61% | 43,849,444 | 88.30% | 1,126,726 | 2.15% |
| NH7 | 48,344,472 | 43,643,127 | 90.28% | 42,541,001 | 88.00% | 1,114,800 | 2.11% |
| NH8 | 57,237,408 | 51,927,544 | 90.72% | 50,648,596 | 88.49% | 1,118,837 | 2.12% |
| NH9 | 52,297,148 | 47,326,784 | 90.50% | 46,101,722 | 88.15% | 1,148,472 | 2.16% |
| NH10 | 52,883,940 | 47,910,552 | 90.60% | 46,791,715 | 88.48% | 1,148,895 | 2.31% |
| NH11 | 46,711,728 | 42,280,563 | 90.51% | 41,246,799 | 88.30% | 1,051,659 | 2.09% |
| NH12 | 39,971,980 | 36,376,795 | 91.01% | 35,508,205 | 88.83% | 1,140,261 | 2.24% |
| NL1 | 56,905,856 | 51,881,338 | 91.17% | 50,634,963 | 88.98% | 1,182,561 | 2.22% |
| NL2 | 38,570,334 | 34,902,615 | 90.49% | 33,984,910 | 88.11% | 1,348,525 | 2.35% |
| NL3 | 59,704,270 | 53,857,045 | 90.21% | 52,452,468 | 87.85% | 1,364,907 | 2.35% |
| NL4 | 58,073,958 | 52,707,267 | 90.76% | 51,342,360 | 88.41% | 1,256,489 | 2.15% |
| NL5 | 50,883,482 | 46,212,573 | 90.82% | 45,072,312 | 88.58% | 1,404,577 | 2.35% |
| NL6 | 47,240,118 | 42,995,973 | 91.02% | 42,000,025 | 88.91% | 1,375,457 | 2.26% |
| NL7 | 57,342,132 | 52,012,505 | 90.71% | 50,663,980 | 88.35% | 1,346,635 | 2.20% |
| NL8 | 47,587,498 | 43,069,766 | 90.51% | 42,019,978 | 88.30% | 1,381,989 | 2.15% |
| NL9 | 56,001,866 | 50,681,440 | 90.50% | 49,348,965 | 88.12% | 1,497,191 | 2.30% |
| NL10 | 44,604,806 | 40,623,462 | 91.07% | 39,701,843 | 89.01% | 1,332,475 | 2.38% |
| NL11 | 42,133,804 | 38,517,999 | 91.42% | 37,563,161 | 89.15% | 1,246,375 | 2.19% |
| NL12 | 61,153,788 | 55,837,964 | 91.31% | 54,491,329 | 89.11% | 1,278,948 | 2.23% |
