# Supplementary material for: Decreased eggshell strength caused by impairment of uterine calcium transport coincide with higher bone minerals and quality in aged laying hens
Source: J Anim Sci Biotechnol. 2024 Mar 4;15:37. doi: 10.1186/s40104-023-00986-2 (PMC10910863; doi:10.1186/s40104-023-00986-2)
Supplement: Supplementary file 5 — Additional file 5: Fig. S1. Quantitative PCR (qPCR) validation of RNA sequencing (RNA-Seq) results. a The initiation stage of eggshell calcification; b The growth stage of eggshell calcification. Asterisks (*) denotes significance (FDR < 0.05 in the RNA-seq, P < 0.05 in the qPCR). CD3E, CD3e molecule; ITGB2, integrin subunit beta 2; CARD11, caspase recruitment domain family member 11; MZB1, marginal zone B and B1 cell specific protein; NCKAP1L, NCK associated protein 1 like; NRG1, neuregulin 1; PTPRC, protein tyrosine phosphatase, receptor type C; SPI1, Spi-1 proto-oncogene; SYK, spleen associated tyrosine kinase; BCL2L14, BCL2 like 14; CA2, carbonic anhydrase 2; CALB1, calbindin 1; CAPN6, calpain 6; ATP2B1, ATPase plasma membrane Ca2+ transporting 1; ATP2B2, ATPase plasma membrane Ca2+ transporting 2; ITPR1, inositol 1,4,5-trisphosphate receptor type 1; SLC8A1, solute carrier family 8 member A1; SLC8A3, solute carrier family 8 member A3; SLC4A4, solute carrier family 4 member 4; SLC26A9, solute carrier family 26 member 9; CLCN5, chloride voltage-gated channel 5; ATP2A2, ATPase sarcoplasmic/endoplasmic reticulum Ca2+ transporting 2. [file 40104_2023_986_MOESM5_ESM.docx]

**Additional file 5**


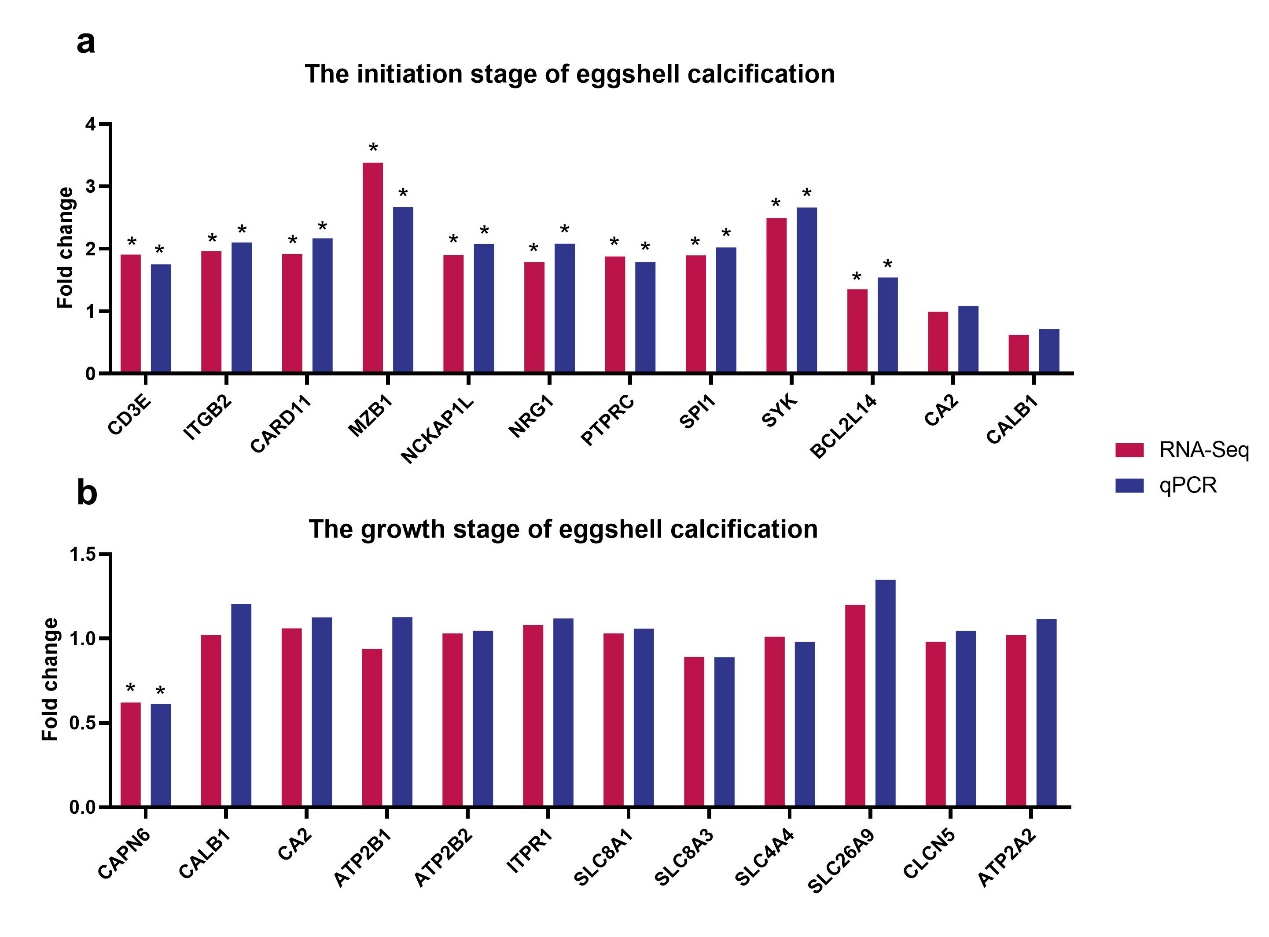
 **Fig. S1** Quantitative PCR (qPCR) validation of RNA sequencing (RNA-Seq) results. **a** The initiation stage of eggshell calcification; **b** The growth stage of eggshell calcification. Asterisks (*) denotes significance (FDR < 0.05 in the RNA-seq, *P* < 0.05 in the qPCR). CD3E, CD3e molecule; ITGB2, integrin subunit beta 2; CARD11, caspase recruitment domain family member 11; MZB1, marginal zone B and B1 cell specific protein; NCKAP1L, NCK associated protein 1 like; NRG1, neuregulin 1; PTPRC, protein tyrosine phosphatase, receptor type C; SPI1, Spi-1 proto-oncogene; SYK, spleen associated tyrosine kinase; BCL2L14, BCL2 like 14; CA2, carbonic anhydrase 2; CALB1, calbindin 1; CAPN6, calpain 6; ATP2B1, ATPase plasma membrane Ca^2+^ transporting 1; ATP2B2, ATPase plasma membrane Ca^2+^ transporting 2; ITPR1, inositol 1,4,5-trisphosphate receptor type 1; SLC8A1, solute carrier family 8 member A1; SLC8A3, solute carrier family 8 member A3; SLC4A4, solute carrier family 4 member 4; SLC26A9, solute carrier family 26 member 9; CLCN5, chloride voltage-gated channel 5; ATP2A2, ATPase sarcoplasmic/endoplasmic reticulum Ca^2+^ transporting 2
