# Supplementary material for: Decreased eggshell strength caused by impairment of uterine calcium transport coincide with higher bone minerals and quality in aged laying hens
Source: J Anim Sci Biotechnol. 2024 Mar 4;15:37. doi: 10.1186/s40104-023-00986-2 (PMC10910863; doi:10.1186/s40104-023-00986-2)
Supplement: Supplementary file 6 — Additional file 6: Table S4. Analysis of differentially expressed genes (|fold change| > 1.3 at a false discovery rate < 0.5) in association with apoptosis and calcium transport. [file 40104_2023_986_MOESM6_ESM.docx]

**Additional file 6**

**Table S4** Analysis of differentially expressed genes (|fold change| > 1.3 at a false discovery rate < 0.5) in association with apoptosis and calcium transport

| **Gene** | **Fold change** | **Annotation** |
| --- | --- | --- |
| Apoptosis | | |
| *BCL2L14* | 1.35 | BCL2 like 14, apoptosis facilitator |
| *C1QA* | 1.57 | Complement C1q A chain, in association with apoptotic cell recognition and removal |
| *C1QB* | 1.54 | Complement C1q B chain, in association with apoptotic cell recognition and removal |
| *CARD11* | 1.92 | Caspase recruitment domain family member 11, regulation of apoptotic process |
| *CD3E* | 1.91 | CD3e molecule, apoptotic signaling pathway |
| *HCLS1* | 1.79 | Hematopoietic cell-specific Lyn substrate 1, negative regulation of leukocyte apoptotic process |
| *IL2RB* | 1.95 | Interleukin 2 receptor subunit beta, negative regulation of apoptotic process |
| *NCKAP1L* | 1.90 | NCK associated protein 1 like, membrane-associated apoptosis protein |
| *NFKBIA* | 1.40 | NFKB inhibitor alpha, cell growth and cell death pathways |
| *PRKCB* | 1.90 | Protein kinase C beta, regulation of endothelial cell proliferation and apoptosis |
| *SYK* | 2.49 | Spleen associated tyrosine kinase, apoptosis gene, participate in the initiation and/or execution of BCR-mediated apoptosis |
| *TNFAIP8L1* | 1.70 | TNF alpha induced protein 8 like 1, related to apoptosis, autophagy and other diseases |
| Calcium transport | | |
| *EDN2* | 1.46 | Endothelin 2, positive regulation of cytosolic calcium ion concentration |
| *IKZF1* | 2.37 | IKAROS family zinc finger 1, positive regulation of cytosolic calcium ion concentration |
| *PRKCB* | 1.90 | Protein kinase C beta, cellular calcium ion homeostasis |
| *UBASH3B* | 1.51 | Ubiquitin associated and SH3 domain containing B, regulation of release of sequestered calcium ion into cytosol |
| *LCP1* | 2.12 | Lymphocyte cytosolic protein 1, calcium ion binding |
| *MGP* | 1.47 | Matrix Gla protein, calcium ion binding |
| *SLC24A4* | 2.32 | Solute carrier family 24 member 4, sodium/potassium/calcium exchanger |
