# Supplementary material for: Decreased eggshell strength caused by impairment of uterine calcium transport coincide with higher bone minerals and quality in aged laying hens
Source: J Anim Sci Biotechnol. 2024 Mar 4;15:37. doi: 10.1186/s40104-023-00986-2 (PMC10910863; doi:10.1186/s40104-023-00986-2)
Supplement: Supplementary file 7 — Additional file 7: Table S5. Comparison of ultrastructural variations in eggshell mammillary layer of the hens laying eggs with different eggshell breaking strength. [file 40104_2023_986_MOESM7_ESM.docx]

**Additional file 7**

**Table S5** Comparison of ultrastructural variations in eggshell mammillary layer of the hens laying eggs with different eggshell breaking strength^1^

| **Item** | **HBS** | **LBS** | ***P*-value** |
| --- | --- | --- | --- |
| Mammillae density | 109.50±21.78 | 101.29±16.95 | 0.414 |
| Confluence | 3.08±0.50 | 3.75±0.90 | 0.089 |
| Type B | 4.08±4.02 | 2.75±2.14 | 0.422 |
| Type A | 1.08±0.15 | 1.12±0.17 | 0.621 |
| Aragonite | 1.08±0.15 | 1.17±0.25 | 0.437 |
| Early fusion | 3.46±0.80 | 2.67±0.85 | 0.075 |
| Late fusion | 4.46±1.10 | 3.42±1.07 | 0.075 |
| Cuffing | 4.79±0.31 | 4.88±0.25 | 0.559 |
| Pitted | 1.67±0.71 | 1.33±0.62 | 0.334 |
| Caps | 1.33±0.36 | 1.50±0.59 | 0.506 |
| Total score | 30.07±4.30 | 28.55±3.08 | 0.432 |

*HBS* High eggshell breaking strength group, *LBS* Low eggshell breaking strength group

^1^Data represent means with standard deviation based on 12 replicates with 25 eggs each
